# The Long Pentraxin 3 Plays a Role in Bone Turnover and Repair

**DOI:** 10.3389/fimmu.2018.00417

**Published:** 2018-03-05

**Authors:** Danka Grčević, Marina Sironi, Sonia Valentino, Livija Deban, Hrvoje Cvija, Antonio Inforzato, Nataša Kovačić, Vedran Katavić, Tomislav Kelava, Ivo Kalajzić, Alberto Mantovani, Barbara Bottazzi

**Affiliations:** ^1^Department of Physiology and Immunology, University of Zagreb School of Medicine, Zagreb, Croatia; ^2^Croatian Institute for Brain Research, University of Zagreb School of Medicine, Zagreb, Croatia; ^3^Humanitas Clinical and Research Center, Milan, Italy; ^4^Oxford BioTherapeutics Ltd., Abingdon, United Kingdom; ^5^Department of Medical Biotechnologies and Translational Medicine, University of Milan, Milan, Italy; ^6^Department of Anatomy, University of Zagreb School of Medicine, Zagreb, Croatia; ^7^Department of Reconstructive Sciences, School of Dental Medicine, UConn Health, Farmingam, CT, United States; ^8^Humanitas University, Milan, Italy; ^9^The William Harvey Research Institute, Queen Mary University of London, London, United Kingdom

**Keywords:** pentraxin 3, fibroblast growth factor 2, inflammation, bone turnover, bone healing, osteoblast, osteoclast

## Abstract

Pentraxin 3 (PTX3) is an inflammatory mediator acting as a fluid-phase pattern recognition molecule and playing an essential role in innate immunity and matrix remodeling. Inflammatory mediators also contribute to skeletal homeostasis, operating at multiple levels in physiological and pathological conditions. This study was designed to investigate the role of PTX3 in physiological skeletal remodeling and bone healing. Micro-computed tomography (μCT) and bone histomorphometry of distal femur showed that PTX3 gene-targeted female and male mice (*ptx3^−/−^*) had lower trabecular bone volume than their wild-type (*ptx3^+/+^*) littermates (BV/TV by μCT: 3.50 ± 1.31 vs 6.09 ± 1.17 for females, *p* < 0.0001; BV/TV 9.06 ± 1.89 vs 10.47 ± 1.97 for males, *p* = 0.0435). In addition, μCT revealed lower trabecular bone volume in second lumbar vertebra of *ptx3^−/−^* mice. PTX3 was increasingly expressed during osteoblast maturation *in vitro* and was able to reverse the negative effect of fibroblast growth factor 2 (FGF2) on osteoblast differentiation. This effect was specific for the *N*-terminal domain of PTX3 that contains the FGF2-binding site. By using the closed transversal tibial fracture model, we found that *ptx3^−/−^* female mice formed significantly less mineralized callus during the anabolic phase following fracture injury compared to *ptx3^+/+^* mice (BV/TV 17.05 ± 4.59 vs 20.47 ± 3.32, *p* = 0.0195). Non-hematopoietic periosteal cells highly upregulated PTX3 expression during the initial phase of fracture healing, particularly CD51^+^ and αSma^+^ osteoprogenitor subsets, and callus tissue exhibited concomitant expression of PTX3 and FGF2 around the fracture site. Thus, PTX3 supports maintenance of the bone mass possibly by inhibiting FGF2 and its negative impact on bone formation. Moreover, PTX3 enables timely occurring sequence of callus mineralization after bone fracture injury. These results indicate that PTX3 plays an important role in bone homeostasis and in proper matrix mineralization during fracture repair, a reflection of the function of this molecule in tissue homeostasis and repair.

## Introduction

The long pentraxin 3 (PTX3) is a member of the evolutionarily conserved pentraxin superfamily, acting as a soluble pattern recognition molecule (1). The mature protein is a complex octameric molecule whose composing protomer subunits comprise a long *N*-terminal domain, unrelated to any known protein, and a *C*-terminal domain homologous to the classical pentraxins, C-reactive protein, and serum amyloid P component (SAP), which collectively form the short arm of the superfamily (2). PTX3 is induced by primary pro-inflammatory cytokines, mainly tumor necrosis factor-α (TNF-α) and interleukin-1 (IL-1), and Toll-like receptor (TLR) agonists in different cell types, such as myeloid (i.e., mononuclear phagocytes, neutrophils, and dendritic cells), endothelial, and stromal cells (1, 3, 4).

A number of animal models and human studies have shown that PTX3 exerts complex non-redundant roles in the innate resistance to pathogens (5–7), in the regulation of inflammation and tissue repair (8), acting also as an oncosuppressor gene (9). The multifaceted biology of PTX3 likely originates from its capacity to interact with a broad spectrum of ligands, including complement components (C1q, factor H, C4 binding protein, ficolins, and mannose-binding lectin), adhesion molecules (P-selectin), growth factors [fibroblast growth factor 2 (FGF2) and other members of the FGF family], and extracellular matrix components (inter-α-trypsin inhibitor, TNF-inducible gene-6, and fibrin), as well as to recognize late apoptotic cells and microbes (1, 4).

Several lines of evidence indicate that pentraxins, through the interplay with various hormones, growth factors, and matrix proteins, are involved in tissue remodeling in both physiological and pathological conditions. It has been shown that *ptx3^−/−^* female mice display a severely impaired fertility due to defective assembly of the viscoelastic hyaluronic acid-rich matrix surrounding the oocyte in the preovulatory cumulus oophorus complex (10, 11). In different models of tissue damage (skin wound healing, chemically induced sterile liver and lung injury, arterial thrombosis), PTX3 deficiency has been associated with increased clot formation, fibrin persistence, and enhanced collagen deposition (12). In this context, macrophages and mesenchymal cells release PTX3 in response to TLR activation and IL-1 stimulation, and the secreted protein promotes remodeling of the fibrin-rich inflammatory matrix, ensuring hemostasis and efficient wound healing. This information notwithstanding, no evidence is available regarding the role of PTX3 in bone turnover, an additional example of tissue remodeling.

Skeletal metabolism is regulated by the cooperative action of two major cell lineages, the mesenchymal osteoblast lineage that mediates bone formation and the hematopoietic osteoclast lineage responsible for bone resorption, with additional contributions from monocytes, macrophages, lymphocytes, and mast cells (13, 14). Bone remodeling is a highly coordinated process consisting of timely occurring bone resorption and formation, triggered by changes in mechanical forces and/or microdamage and modulated by a number of factors including inflammation, hormonal levels, and local mediators (15). At various stages of bone remodeling and repair, progenitor cells, osteoclasts, and osteoblasts communicate through multiple cell-to-cell and cell-to-matrix interactions *via* a number of signaling molecules, including tissue growth factors, cytokines, and matrix proteins. Maintenance of skeletal homeostasis during physiological bone turnover and initiation of efficient reparative process after bone injury are crucial to preserve the major biological roles of the bone tissue, i.e., provide support to mechanical load and exert metabolic functions (16–18).

Bone remodeling is crucial to control the reshaping or replacement of bone during growth and following injuries (15). Fractures are one of the most frequent injuries of the musculoskeletal system, affecting not only the bone itself but also the periosteum, blood vessels, and the surrounding soft tissue. Fracture healing is a sequential process that requires a complex interaction among molecular factors, immune cells, resident tissue cells, and progenitor cells to stimulate inflammation, angiogenesis, mesenchymal progenitor recruitment, cartilage and bone formation, extracellular matrix synthesis, and callus remodeling (19, 20). Inflammatory cells (neutrophils and macrophages) infiltrate the injured area, where they produce inflammatory mediators, such as TNF-α, IL-6, and prostaglandin E2. Mesenchymal progenitors recruited to the site of injury undergo endochondral bone formation, initially differentiating into cartilage, which is further replaced by the calcified bone that incorporates the bone marrow. In addition, periosteal mesenchymal progenitors adjacent to the fracture gap differentiate directly into osteoblasts by intramembranous ossification. Therefore, a local acute inflammatory response sets within the first few days from fracture is required to initiate an adequate healing process (21, 22).

The aim of this study is to investigate the role of the inflammatory mediator PTX3 during physiological bone remodeling and fracture healing. By using *ptx3^−/−^* mice, we found that PTX3 is important for bone tissue homeostasis and fracture repair. PTX3 is expressed by osteoblast lineage cells, with osteoprogenitor cells being the major source of PTX3 in the early inflammatory stages of fracture healing.

## Materials and Methods

### Mice

*Ptx3^−/−^* (knockout) mice, generated as previously described (6), were used on a 129/SvPas or C57BL/6J inbred (backcrossed for 11 generations) genetic background. Wild-type (*ptx3^+/+^*) mice were obtained from Charles River (Charles River Laboratories, Calco, Italy) or were cohoused littermates. Femora and lumbar vertebrae were collected from mice housed in the specific pathogen-free animal facility of the Humanitas Clinical and Research Center in individually ventilated cages. Animals of 10–12 weeks old (young mice) or 6–8 months old (old mice) were used. Procedures involving animals handling and care were conformed to protocols approved by the Ethics Committees of the Humanitas Clinical and Research Center (Rozzano, Milan, Italy). For the *in vivo* fracture experiments, the mice were transported from Humanitas Clinical and Research Center and housed until 14–16 weeks old, in groups of three to four per cage, and maintained at standard conditions with food and water *ad libitum* at the animal facility of the Croatian Institute for Brain Research, University of Zagreb School of Medicine (Zagreb, Croatia). All experimental procedures were reviewed and approved by the Ethics Committee of the University of Zagreb School of Medicine and conducted in accordance with accepted standards of humane care and use of laboratory animals. All procedures were performed under tribromoethanol (Avertin) anesthesia, and all efforts were made to minimize suffering of mice during experiments.

### Recombinant PTX3

Recombinant human PTX3 and its *C*-terminal (C-PTX3) and *N*-terminal (N-PTX3) domains were purified under endotoxin-free conditions from the supernatants of stably transfected Chinese hamster ovary cells as previously described (8).

### Bone Cell Cultures

For osteoblast generation, bone marrow cells were flushed out from the femoral medullary cavity of *ptx3^+/+^* or *ptx3^−/−^* mice. Cells adjacent to endosteal and periosteal surfaces of mid-diaphyseal femoral bone shafts were released by homogenization. Harvested cells were plated into 24-well plates at a density of 0.8 × 10^6^ cells/well in 0.8 mL/well of α-MEM/10% fetal calf serum (FCS). Three wells from two to three mice were done for each gender/genotype, and three independent experiments were performed. Osteoblast differentiation was induced from day 7 by addition of 50 µg/mL ascorbic acid (AA) and 8 mM β-glycerophosphate (β-GP) (Sigma-Aldrich for both, Saint Louis, MO, USA). At day 14–17, osteoblast cultures were stained cytochemically for the activity of alkaline phosphatase (ALP) using a commercially available kit (Sigma-Aldrich), and red color ALP^+^ osteoblast surface per well was measured by a custom-made software (23). Total colony surface was assessed by methylene blue staining.

For osteoclast generation, bone marrow cells from *ptx3^+/+^* or *ptx3^−/−^* mice were cultured overnight with 5 ng/mL recombinant mouse M-CSF (R&D Systems, NE Minneapolis, MN, USA) in α-MEM/10% FCS to stimulate the monocyte/macrophage lineage followed by harvesting of non-adherent cells as enriched hematopoietic monocyte/macrophage progenitors. Non-adherent cells were replated into 48-well plates at a density of 0.25 × 10^6^/well in 0.5 mL/well of α-MEM/10% FCS supplemented with 20 ng/mL M-CSF and 40 ng/mL RANKL (R&D Systems). Spleen cells, obtained by smashing the spleen tissue between a pair of frosted microscope slides, were cultured in 48-well plates at a density of 0.5 × 10^6^/well in 0.5 mL/well of α-MEM/10% FCS supplemented with 20 ng/mL M-CSF and 40 ng/mL RANKL. At day 5–9 of culture, tartrate-resistant acid phosphatase (TRAP)^+^ multinucleated cells (≥3 nuclei/cell) were identified using a commercially available kit (Sigma-Aldrich) and counted by light microscopy (24). Four wells from two to three mice were done for each gender/genotype, and three independent experiments were performed.

Osteoblastogenic and osteoclastogenic cultures were treated with a dose range of PTX3 (0–50 nM). In some experiments, either full-length PTX3, its *N*- or *C*-terminal domain (50 nM for all), or/and FGF2 (0.25 nM and 0.5 nM) were added to osteoblastogenic cultures in addition to AA and β-GP. Molarity was expressed based on the molecular weight of the protomer subunits of PTX3 and its *N*- and *C*-terminal domains, and the primary sequence of FGF2, that is, 42.500 kDa for PTX3, 18.163 kDa for N-PTX3, 25.000 kDa for C-PTX3, and 17.254 kDa for FGF2 (25).

### Flow Cytometry

For flow cytometric analysis, bone marrow and spleen cells were harvested as described previously in Ref. (24). Erythrocytes were lysed with red blood cell lysing buffer (Sigma-Aldrich), and single-cell suspensions were obtained by filtering through a 100-µm Nytex mesh. Cells were counted in a hemocytometer by trypan blue exclusion and labeled using a mix of commercially available monoclonal antibodies against hematopoietic/endothelial markers (anti-CD45 APC, clone 30-F11; anti-Ter119 APC, clone TER-119; anti CD31 APC, clone 390), lymphoid lineage markers (anti-CD3 FITC, clone 145-2C11; anti-B220 FITC or PE-Cy7, clone RA3-6B2; anti-NK1.1 FITC or APC; clone PK136), myeloid lineage markers (anti-CD11b APC-eFluor 780, clone M1/70; anti-Ly6C APC, clone HK1.4; anti-Ly6G PerCP-eFluor 710, clone 1A8), osteoclast progenitor markers (anti-CD115/cFms biotinylated, clone AFS98; anti-CD117/cKit APC, clone 2B8), and osteoblast progenitor markers (anti-CD166/ALCAM PE, clone eBioALC48; anti-Sca-1 FITC, clone D7), all from eBiosciences (San Diego, CA, USA). Suspensions were incubated on ice for 40 min, followed by washing in staining medium [2% FCS in phosphate-buffered saline (PBS)]. As a second step (for biotinylated anti-CD115), cells were stained with streptavidin coupled to PE-Cy7 on ice for 40 min. Finally, cells were resuspended in staining medium containing 0.25 μg/tube 7-aminoactinomycin D or 4’,6-diamidino-2-phenylindole for dead cell exclusion. Gates were defined using unlabeled cells, cells labeled by isotype controls, and fluorescence minus one controls. Samples were acquired using Attune (Life Technologies, ABI, Carlsbad, CA, USA) or BD FACSAria I (BD Biosciences, San Jose, CA, USA) instrument and analyzed by FlowJo software (TreeStar, Ashland, OR, USA).

Fluorescence-activated cell sorting (FACS) of osteoprogenitor cells was performed in a BD FACSAria I instrument. Cells were extracted from the periosteal layer and callus tissue and labeled as described previously (24). Briefly, tibias were dissected, soft tissue was removed, and bone marrow was flushed. Periosteal layer of diaphyseal bone fragments/fracture callus were briefly crushed using tissue homogenizer and further digested in PBS containing 0.1% collagenase A (Roche, Mannheim, Germany) for 30 min at 37°C with agitation. For phenotyping of osteoblast lineage cells, the following antibodies were used: anti-CD51 biotinylated (clone RMV-7; with streptavidin coupled to PE-Cy7), anti-CD105 PE or PE-Cy7 (clone MJ7/18), anti-CD140b APC (clone APB5), anti-α-smooth muscle actin (αSma) Alexa Fluor 488 (clone 1A4), and anti-Sca-1 FITC or Pacific Blue (clone D7), according to previous studies (26). Samples were acquired at a speed of approximately 3,000 cells/s to separate hematopoietic/endothelial-negative (CD45*^−^*Ter119*^−^*CD31*^−^*) and hematopoietic-positive fractions (CD45^+^Ter119^+^CD31^+^). Sorting parameters and experimental set-up were optimized for high purity sorting. Sorting purity was determined by a reanalysis of fractioned populations and was greater than 98% for all samples.

For flow cytometric analysis of PTX3 expression, purified goat immunoglobulin G (IgG) anti-mouse PTX3 (R&D Systems) or purified IgG from pre-immune goat serum (Agilent-Dako, Santa Clara, CA, USA) were used with anti-goat antibody coupled to PE (eBiosciences) as previously described (9). Cells were first stained for surface marker expression using the previously described antibodies to hematopoietic and osteoprogenitor lineages, then permeabilized with BD Cytofix/Cytoperm kit (BD Biosciences), according with manufacturer’s instructions, and, finally, stained for intracellular expression of PTX3. Gates were defined using unlabeled cells and isotype controls. Specificity of anti-PTX3 labeling was also confirmed by parallel staining in *ptx3^+/+^* and *ptx3^−/−^* mice. Samples were acquired using BD FACSAria I instrument and analyzed by FlowJo software.

### Fractures

Closed transverse diaphyseal fractures of tibias were created in 14- to 16-week-old female *ptx3^+/+^* and *ptx3^−/−^* mice. Anesthesia was induced and maintained using tribromoethanol (Avertin), and 0.08 mg/kg buprenorphine hydrochloride was administered subcutaneously for analgesia. Prior to fracture, a tract was created through the tibial plateau into the medullary canal using a 25-gauge needle, through which a 0.4-mm diameter stainless steel pin was positioned to fix the subsequent fracture (26). Fractures were created 1–2 mm proximal to the distal tibia-fibula junction using the modified, limited displacement three-point contact device (27). Unfractured bones and bones with inserted pins without fracture were used as controls.

### Micro-Computed Tomography

The distal metaphyses of femora and second lumbar vertebrae were scanned using a micro-computed tomography (μCT) system (1172 SkyScan, SkyScan, Kontich, Belgium) at 50 kV and 200 mA with a 0.5 aluminum filter using a detection pixel size of 4.3 µm (24). Images were captured every 0.7° through 180° [second lumbar (L2) vertebrae] and every 0.7° through 360° (femora) rotation. The scanned images were reconstructed using the SkyScan Recon software and analyzed using SkyScan CTAnalyser. Three-dimensional analysis and reconstruction of trabecular bone were performed on the bone region 1–2.3 mm distal to the growth plate. The trabecular bone compartment was manually delineated from the cortical bone, and the following parameters were measured: trabecular bone volume fraction (BV/TV, %), trabecular number (Tb.N/μm), trabecular thickness (Tb.Th; μm), and trabecular separation (Tb.Sp; μm). In fracture experiments, tibial fractured areas were scanned and three-dimensional reconstructions were performed to measure mineralized callus bone volume (BV/TV, %).

### Histology and Histomorphometry

Femora were fixed in 4% paraformaldehyde, demineralized in 14% ethylenediaminetetraacetic acid in 3% formaldehyde, dehydrated in increasing ethanol concentrations, and embedded in paraffin (24). Six-micrometer sections were cut with a Leica SM 2000 R rotational microtome (Lieca SM 2000 R, Leica Biosystems, Nussloch, Germany) and stained with Goldner–Masson trichrome (GT) or TRAP activity. Histomorphometric analysis was performed under Axio Imager microscope (Carl Zeiss) using the OsteoMeasure software (Osteo-Metrics, Decatur, USA).

For static histomorphometry, metaphyseal regions of GT-stained femora, 1 mm distally from the epiphyseal plate, were analyzed under 5× objective magnification. The analyzed variables included trabecular volume (BV/TV), trabecular thickness (Tb.Th, μm), trabecular number (Tb.N/μm), and trabecular separation (Tb.Sp, μm). On TRAP-stained sections, osteoclasts were identified as multinucleated cells placed adjacent to the bone surface. For dynamic histomorphometry, mice were injected twice with calcein, 20 mg/kg each, at day 7 and 2 prior to sacrifice. Seven-micrometer sections of undecalcified femora were cut using Leica cryostat and analyzed under a fluorescent microscope (Axio Imager, Carl Zeiss); mineral apposition rate (μm/day or %/day) and bone formation rate (BFR; μm^3^/μm^2^/day) were automatically calculated.

### Immunohistochemistry

Bone sections were prepared as described and deparaffinized. Fractured bones had the intramedullary pin removed prior to embedding. Sections were incubated overnight at 65°C with 10 mM sodium citrate (pH 6) for antigen retrieval. Endogenous peroxidases were inactivated with 0.03% H_2_O_2_ in methanol for 30 min at room temperature. Serial sections were blocked with Rodent block M (Bio care Medical, Concord, CA, USA) for PTX3, osterix (Osx), and runt-related transcription factor 2 (Runx2) staining or 5% BSA in 0.5% Triton/PBS for FGF2 staining and then incubated with the primary antibody at 4°C overnight. As primary antibodies, we used an affinity purified rabbit IgG against human PTX3 (5 µg/mL), a monoclonal rabbit IgG against mouse/rat/human Osx (1:1,000; Abcam, Cambridge, UK), a monoclonal rabbit IgG against mouse/rat/human Runx2 (1:2,000; Abcam), or polyclonal goat IgG against human FGF2 (5 µg/mL; R&D Systems) (28). Secondary reagents were Envision anti-rabbit horse radish peroxidase (HRP) (Dako, Glostrup, Denmark) for PTX3, Osx, and Runx2 staining or goat probe with goat HRP polymer (Bio care Medical) for FGF2 staining. Reactions were visualized using 3.3′-diaminobenzidine as a chromogen (Vector laboratories Inc. Burlingham, CA, USA) and stopped with distilled water. Sections were counterstained with hematoxylin and covered using Hydromount (National Diagnostics, Atlanta, GA, USA).

For quantitative analysis of PTX3 expression, images of three serial sections from four mice per group were digitally captured at 200× magnification. PTX3 immunoreactive areas were measured using ImageJ software (version 1.45; NIH, MD, USA). Immunoreactive area was automatically selected on the basis of immunoreactive sample intensity, and results are expressed as median percentage of the immunoreactive pixels in total pixels with interquartile range (IQR).

### Gene Expression Analysis

Total RNA from cultures and callus-derived cells was extracted using TRIzol Reagent (Applied Biosystems, Thermo Fisher Scientific, Waltham, MA, USA), reversely transcribed (1 µg) to cDNA, and amplified (20 ng/well in duplicates) by quantitative (q)PCR using an AB7500 (Applied Biosystems) instrument. Each sample was prepared by pooling three to four culture wells or callus tissue from two to three bones. RNA quality was verified on a Bioanalyzer RNA PicoChip (Agilent Technologies, Santa Clara, CA, USA) and quantified using Nanodrop spectrophotometer (Thermo Scientific, Thermo Fisher Scientific). Expression of osteoblast-specific genes: osteocalcin (OC), bone sialoprotein (BSP), Osx, ALP, α2-chain of type I collagen (Col1); osteoclast specific genes: colony stimulating factor 1 receptor (cFms), receptor activator of nuclear factor-κB (RANK), and calcitonin receptor (CalcR); inflammatory markers: C-C chemokine ligand 2 (CCL2), TNF-α, IL-1α, and IL-6; and regulatory molecules: PTX3 and FGF2 was determined using commercially available TaqMan Gene Expression Assays (Applied Biosystems). The expression of a specific gene was calculated according to the relative standard curve of gene expression in the calibrator sample (cDNA from *ptx3^+/+^* callus or cultures) and then normalized to the expression level of the β-actin gene as an endogenous control (24).

### ELISA

Blood was collected from the retro-orbital plexus of control and fractured mice at day 2 and 6 postfracture; serum was stored at −20°C until cytokine analysis. Murine PTX3, CCL2, TNF-α, and IL-6 levels were measured in serum by ELISA (DuoSet ELISA Development System, R&D Systems) according to manufacturer’s instructions.

### Statistics

Normality of data distribution was verified using Kolmogorov–Smirnov test. Number of independent experiments performed for each setting with total number of analyzed mice per group is indicated in figure legends. Results are presented as mean ± SD for normally distributed data or median with IQR for not-normally distributed data. Statistical analysis of the group difference was performed using the parametric Student’s *t*-test (for group-to-group comparison) and analysis of variance with Student–Newman–Keuls *post hoc* test (for multiple group comparisons) or the non-parametric Kruskal–Wallis test followed by Mann–Whitney test. For all experiments, α-level was set at 0.05. Statistical analysis was performed using the software packages MedCalc (version 12.5, MedCalc Inc., Mariakerke, Belgium).

## Results

### *Ptx3^−/−^* Female Mice Have Lower Bone Mass Compared to *ptx3^+/+^* Mice

**Figure 1:**
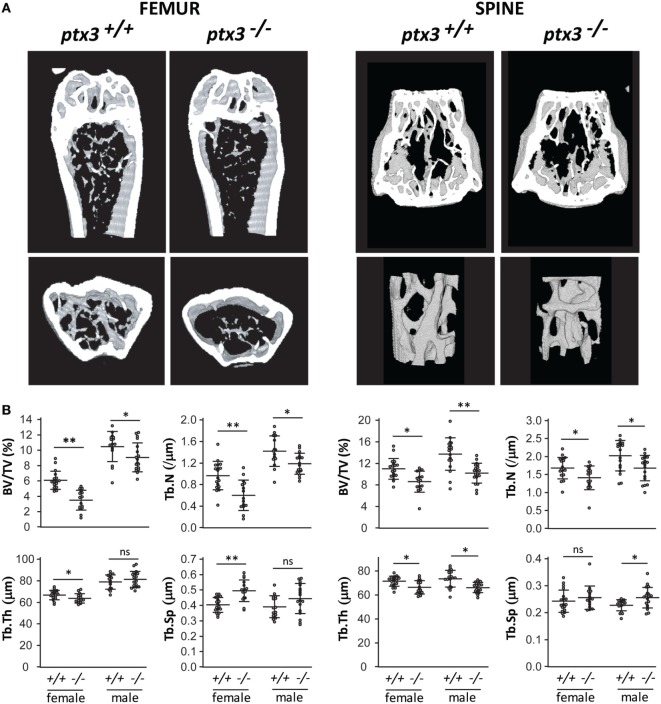
Bone phenotype of the axial and appendicular skeleton in young adult *ptx3^+/+^* and *ptx3^−/−^* mice. Female and male *ptx3^+/+^* and *ptx3^−/−^* mice (10–12 weeks of age) were sacrificed, and the second lumbar vertebrae and distal femora were analyzed for trabecular bone parameters by micro-computed tomography (μCT). **(A)** Representative images of three-dimensional reconstruction of selected areas of the distal femur (left panel) and second lumbar vertebra (right panel). **(B)** Quantitative μCT analysis of trabecular bone volume (BV/TV, bone volume/total volume), trabecular number (Tb.N), trabecular thickness (Tb.Th), and trabecular separation (Tb.Sp) in the distal femoral metaphysis (left panel) and second lumbar vertebra (right panel). Cumulative data from three independent sets of experiments are shown (*n* = 18–20 mice per group). Dots represent individual mice, and horizontal lines and error bars are mean ± SD; statistically significant difference between corresponding *ptx3^+/+^*and *ptx3^−/−^* groups is marked on plots (*p < 0.05, **p < 0.001; unpaired Student’s *t*-test); ns, not significant.

In the first set of experiments, we aimed to investigate the role of PTX3 in maintenance of skeletal homeostasis. Both axial (at the level of L2 vertebra) and appendicular (at the level of distal femoral metaphysis) skeleton of *ptx3^+/+^*and *ptx3^−/−^* mice were assessed by μCT. Distal femora from young adult *ptx3^−/−^*females (10–12 weeks old) on B6 background showed highly significant decrease in trabecular bone volume (*p* < 0.0001), which was paralleled by decreased trabecular number and trabecular thickness, as well as increased trabecular separation compared to *ptx3^+/+^* mice (Figure 1). Difference in distal femoral trabecular bone volume was marginally significant between *ptx3^−/−^* and *ptx3^+/+^* males (*p* = 0.0435), with lower trabecular number in *ptx3^−/−^*mice. Moreover, trabecular bone volume, trabecular number, and trabecular thickness were decreased in lumbar vertebrae from *ptx3^−/−^* compared to *ptx3^+/+^* female and male mice (Figure 1). Histomorphometry of distal femoral histological sections confirmed lower bone mass in *ptx3^−/−^* female and male mice found by μCT (Figure S1 in Supplementary Material). Finally, we analyzed bone mass in aged female *ptx3^−/−^* mice (6–8 months old) on B6 background and found significantly lower distal femoral trabecular bone volume and trabecular thickness compared to *ptx3^+/+^* mice (Figure S2 in Supplementary Material).

In addition, μCT analysis performed in young adult mice on S129 background revealed the decrease in trabecular bone volume in distal femora of both female and male *ptx3^−/−^*mice as well as in axial skeleton of *ptx3^−/−^* females (Figure S3 in Supplementary Material). These findings point to strain-independent alteration in bone remodeling, in particular in female mice lacking the PTX3 gene.

### PTX3 Deficiency Does Not Affect the Osteoclastogenic and Osteoblastogenic Potential of Progenitor Cells *In Vitro*

**Figure 2:**
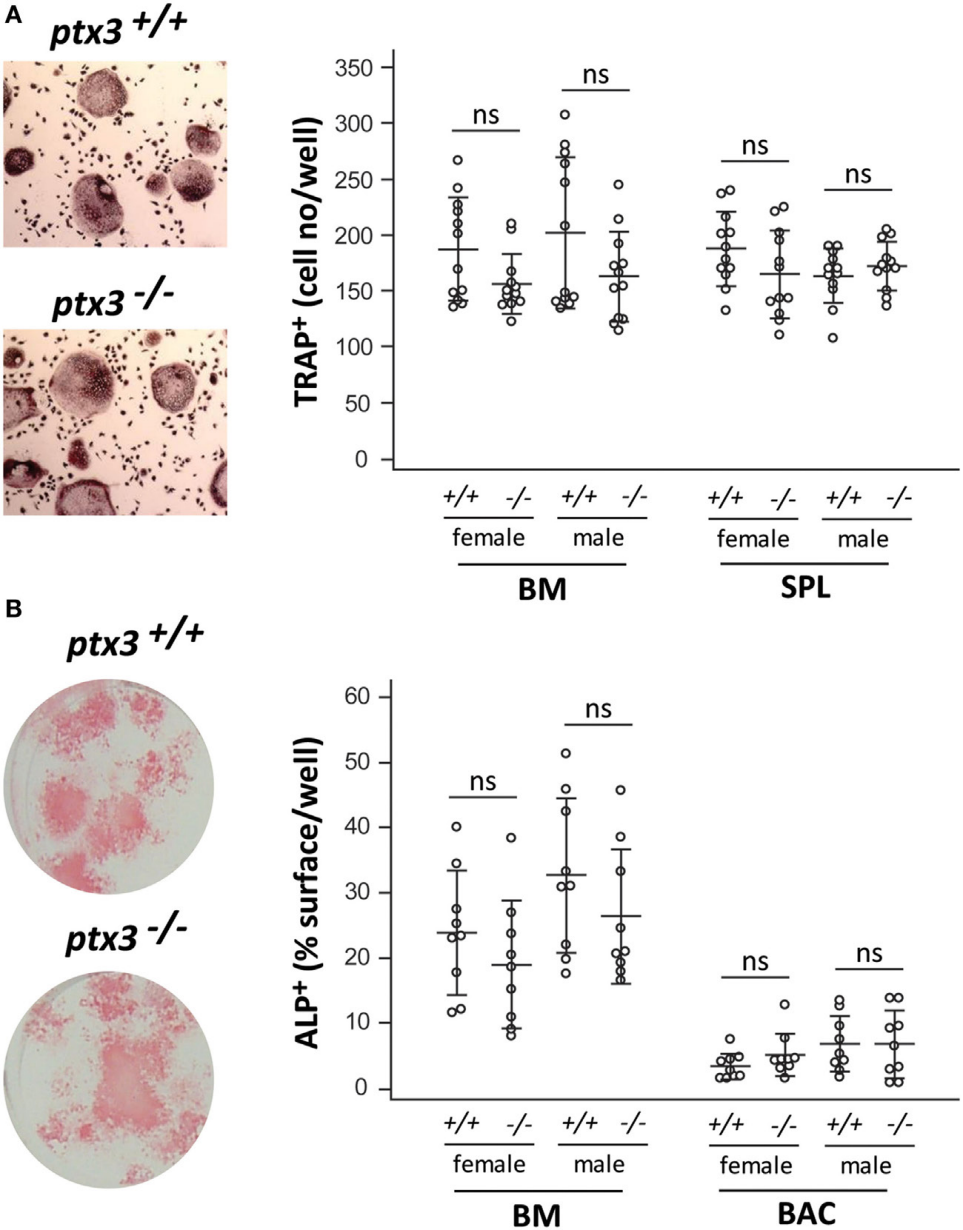
*In vitro* osteoclastogenic and osteoblastogenic potential of *ptx3^+/+^* and *ptx3^−/−^* mice. Bone marrow (BM) and spleen (SPL) cells were extracted from *ptx3^+/+^* and *ptx3^−/−^* female and male mice (10–12 weeks of age) and plated in osteoclastogenic cultures (in the presence of RANKL and M-CSF), or BM and bone-associated cells (BACs) were plated in osteoblastogenic cultures [in the presence of ascorbic acid (AA) and β-GP]. Cells from two to three mice were pooled; four wells for osteoclasts and three wells for osteoblasts were done for each gender/genotype; three independent sets of experiments were performed. **(A)** Representative microphotographs of osteoclasts differentiated *in vitro* from the BM of *ptx3^+/+^* and *ptx3^−/−^* mice, cytochemically stained for tartrate-resistant acid phosphatase (TRAP) activity (left panel). TRAP^+^ cells with three or more nuclei in cultures of BM and spleen from *ptx3^+/+^* and *ptx3^−/−^* female and male mice were counted, and results are expressed as number of TRAP^+^ cells/well (right panel). **(B)** Representative microphotographs of osteoblasts differentiated *in vitro* from BM of *ptx3^+/+^* and *ptx3^−/−^* mice, cytochemically stained for alkaline phosphatase (ALP) activity (left panel). ALP^+^ surface area in cultures of BM and BACs from *ptx3^+/+^* and *ptx3^−/−^* female and male mice were measured, and results are expressed as percentage of ALP^+^ surface/well (right panel). Data from all performed experiments are shown (*n* = 12 wells per group for osteoclasts; *n* = 9 wells per group for osteoblasts). Each dot represents values from one culture well; horizontal lines and error bars are mean ± SD; no statistically significant difference between corresponding *ptx3^+/+^*and *ptx3^−/−^* groups was observed (unpaired Student’s *t*-test).

To clarify the mechanisms underlying the bone phenotype in *ptx3^−/−^* mice, we analyzed the osteoclastogenic and osteoblastogenic potential of bone progenitor cells. Osteoclasts were differentiated from bone marrow and spleen cells in the presence of RANKL and M-CSF. Number of differentiated osteoclasts, detected as TRAP^+^ multinucleated cells, did not significantly differ between *ptx3^+/+^* and *ptx3^−/−^* mice in both females and males (Figure 2A). In addition, frequency of putative osteoclast progenitors (29, 30), defined as CD3*^−^*B220*^−^*CD11b*^−^*^/lo^cKit^+^cFms^+^ subset for bone marrow (Figure S4A in Supplementary Material) and CD3*^−^*B220*^−^*NK1.1*^−^*CD11b^+^Ly6C^+^cFms^+^ subset for spleen (not shown), was comparable between *ptx3^+/+^* and *ptx3^−/−^* mice.

Osteoblasts were differentiated *in vitro* from bone marrow and bone adjacent (endosteal and periosteal) cells by the addition of AA and β-GP. *Ptx3^−/−^* mice did not show significant difference in the percentage of ALP^+^ surface area (Figure 2B) as well as frequency of osteoprogenitor cells (31, 32), defined as CD45*^−^*Ter119*^−^*CD31*^−^*CD166^+^Sca-1^+^ subset (Figure S4B in Supplementary Material for bone marrow; not shown for bone adjacent cells). Our findings indicated that osteoclast and osteoblast progenitors retain similar differentiation potential in the absence of PTX3 *in vitro* and prompted us to focus on the functional properties of bone cells *in vivo*.

### *Ptx3^−/−^* Mice Have Reduced *In Vivo* Functional Activity of Osteoblast Lineage Cells

**Figure 3:**
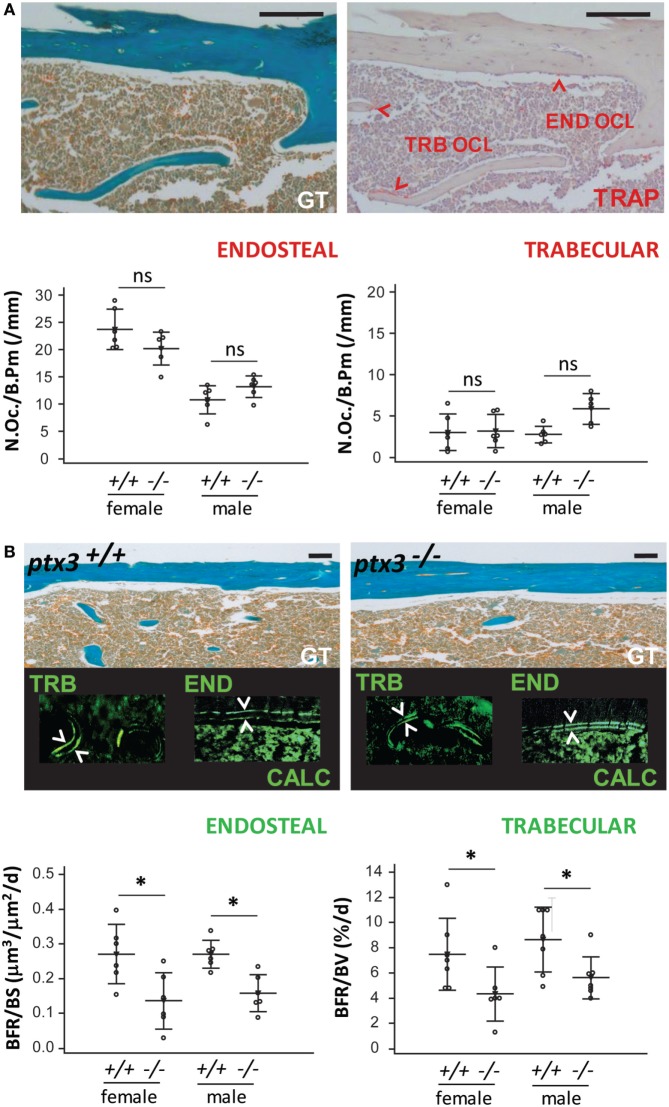
*In vivo* osteoclast and osteoblast activity in *ptx3^+/+^* and *ptx3^−/−^* mice. Sections of distal femoral metaphysis from *ptx3^+/+^* and *ptx3^−/−^* female and male mice (10–12 weeks of age) were histochemically stained for tartrate-resistant acid phosphatase (TRAP) activity. Goldner–Masson trichrome (GT) staining was used to visualize mineralized matrix (green) in serial sections of cortical and trabecular (TRB) compartments (size bar: 100 µm). Calcein (CALC) interlabel distance in distal femoral metaphysis from *ptx3^+/+^* and *ptx3^−/−^* female and male mice were measured to estimate the rate of bone formation. **(A)** Representative microphotographs of active TRAP^+^ osteoclasts (OCL) adjacent to edosteal (END) and TRB bone surfaces (indicated by arrowheads) in *ptx3^+/+^* mice (not shown for *ptx3^−/−^* mice). Quantification of END and TRB TRAP^+^ osteoclasts per bone perimeter (N.Oc./B.Pm) in *ptx3^+/+^* and *ptx3^−/−^* female and male mice (*n* = 6–8 mice per group) is reported below. **(B)** Representative microphotographs of distal femoral metaphyseal sections from mice injected with calcein; interlabel distance visualized by fluorescent microscopy (indicated by arrowheads). Histomorphometric evaluation of endosteal bone formation rate (BFR) relative to bone surface area (BFR/BS) and TRB BFR relative to bone volume (BFR/BV) in *ptx3^+/+^* and *ptx3^−/−^* female and male mice (*n* = 6–8 mice per group) is reported below. Dots represent individual mice, and horizontal lines and error bars are mean ± SD; statistically significant difference between corresponding *ptx3^+/+^*and *ptx3^−/−^* groups is marked on plots (*p < 0.05; unpaired Student’s *t*-test).

Since reduction in bone mass arises from unbalanced bone resorption (by osteoclasts) and formation (by osteoblasts), we assessed *in vivo* activity of osteoclasts and osteoblasts on histology specimens from distal femora. *Ptx3^−/−^* mice did not differ from *ptx3^+/+^* mice in the number of active trabecular and endosteal osteoclasts, counted as multinucleated TRAP^+^ cells adjacent to the bone surface (Figure 3A). However, dynamic histomorphometry, measuring distance of calcein labels injected in 5-day interval, revealed defective osteoblast activity and decreased BFR on endosteal and trabecular bone surface in both female and male *ptx3^−/−^* mice (Figure 3B). These findings suggest that the lower bone volume in *ptx3^−/−^* mice might be ascribed to suppressed osteoblast function and prompted us to exploit functional model of bone injury requiring intensive new bone formation *in vivo*.

### *Ptx3^−/−^* Mice Have Reduced Callus Mineralized Volume during Fracture Healing

**Figure 4:**
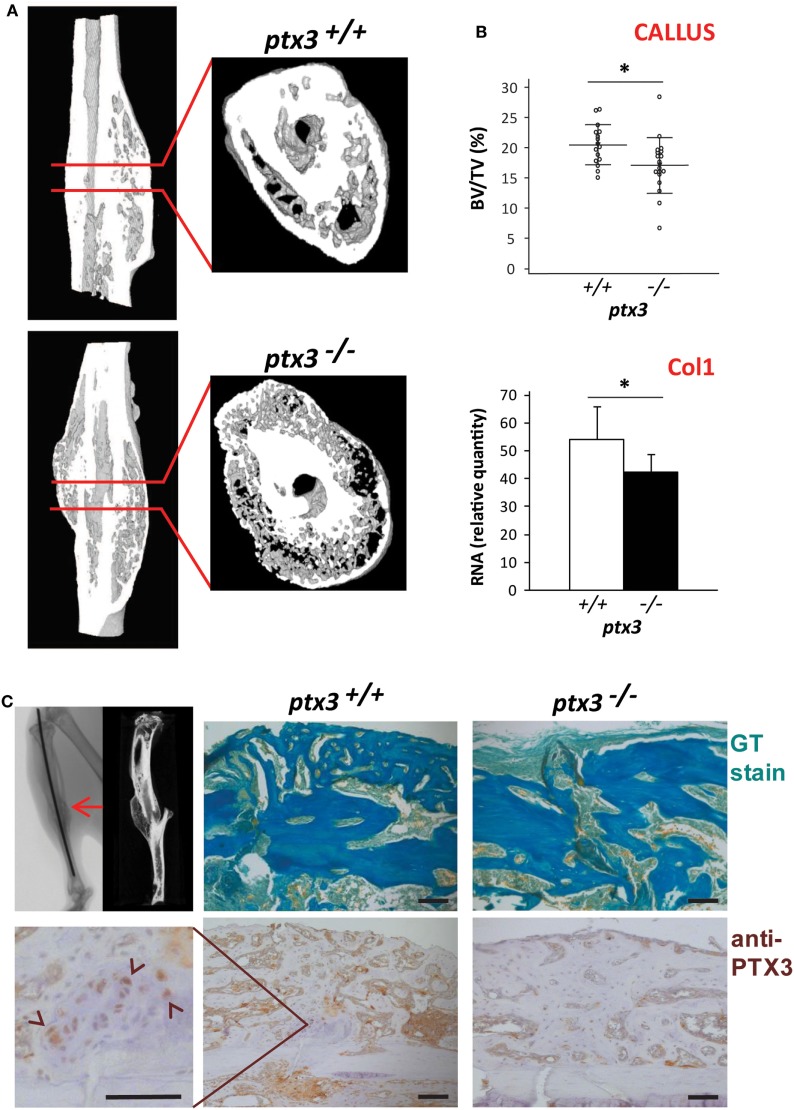
Evaluation of fracture callus tissue at 3 weeks postfracture in *ptx3^+/+^* and *ptx3^−/−^* female mice. Stabilized closed transversal mid-tibial fracture model was applied in female *ptx3^+/+^*(*n* = 16) and *ptx3^−/−^* (*n* = 18) mice on B6 background (14–16 weeks of age). Assessment of callus tissue at 3 weeks postfracture was performed by micro-computed tomography (μCT) analysis, qPCR, and histology. **(A)** Representative microphotographs of three-dimensional reconstruction of tibial fractured areas and callus tissue sections of *ptx3^+/+^* and *ptx3^−/−^* mice assessed by μCT. **(B)** Quantitative μCT analysis of mineralized callus bone volume (BV/TV, bone volume/total volume) of *ptx3^+/+^* and *ptx3^−/−^* mice. Dots represent individual mice, and horizontal lines and error bars are mean ± SD (upper panel). qPCR assessment of bone-specific α2-chain of type I collagen (Col1) expression in *ptx3^+/+^* and *ptx3^−/−^* mice. Values (*n* = 5 per group) are presented as mean ± SD (lower panel). Statistically significant difference between corresponding *ptx3^+/+^*and *ptx3^−/−^* groups is marked on plots (*p < 0.05; unpaired Student’s *t*-test). **(C)** Sections of mid-tibial callus area (indicated by arrow on μCT microphotograph) from *ptx3^+/+^* and *ptx3^−/−^* female mice were stained immunohistochemically with an anti-PTX3 antibody. Goldner–Masson trichrome (GT) staining was used to visualize mineralized matrix (green) in callus compartment (size bar: 100 µm). In the selected area, PTX3^+^ cuboidal osteoblasts present during the anabolic stage of fracture healing are indicated by arrowheads.

A tibia mid-diaphyseal fracture model allowed assessment of the capacity of new bone formation and bone regeneration in *ptx3^−/−^* mice. Three weeks after tibial fracture injury, during the reparative phase of the fracture healing process marked by intensive endochondral and intramembranous ossification, both percentage of mineralized callus tissue and expression of the bone-specific marker Col1 were lower in female *ptx3^−/−^*mice than in *ptx3^+/+^* controls (Figures 4A,B). Immunohistochemistry revealed that PTX3 is present in the callus tissue of *ptx3^+/+^* mice during the mineralizing phase of fracture healing (Figure 4C). Areas of newly formed mineralized tissue (detected as GT green stain) produce PTX3, including cuboidal active osteoblasts, mostly apparent on high magnification. Therefore, we concluded that PTX3 has a role during fracture repair and that inactivation of the PTX3 gene is responsible for the reduced osteoblast function and incomplete bone formation observed in *ptx3^−/−^* mice.

### PTX3 Is Expressed during the Early Inflammatory Phase of Bone Healing

**Figure 5:**
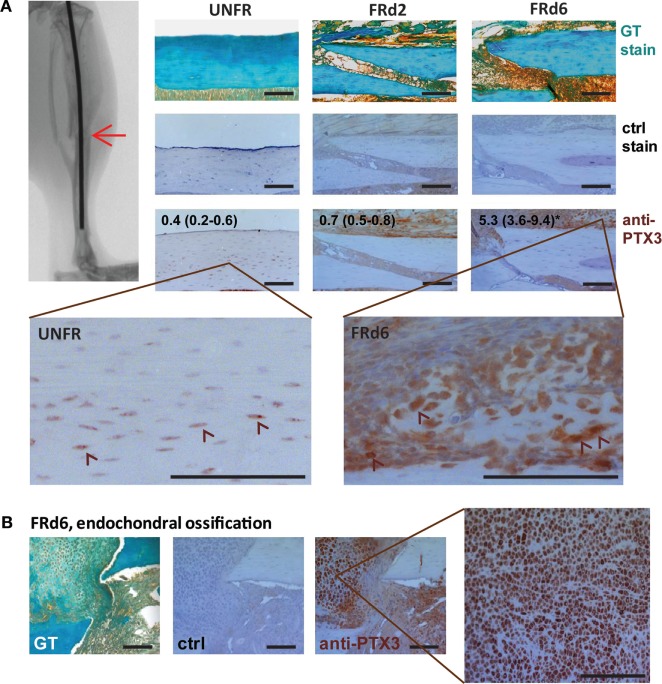
Pentraxin 3 (PTX3) expression within the fractured areas at early postfracture days in *ptx3^+/+^* female mice. A stabilized closed transversal mid-tibial fracture model was applied in female *ptx3^+/+^* (*n* = 18) mice (14–16 weeks of age). Callus tissue was assessed at 2 (FRd2) and 6 (FRd6) days postfracture (indicated by arrow on micro-computed tomography). **(A)** Sections of mid-tibial unfractured bone (UNFR) and fracture area (FRd2 and FRd6) from *ptx3^+/+^* female mice were stained immunohistochemically with an anti-PTX3 antibody. Numbers indicate median percentage of immunopositive area (immunopositive pixels/total pixels) with interquartile range analyzed by ImageJ software (*n* = 4 slides per group); statistically significant difference was observed for FRd6 compared to other groups (*p < 0.05; Kruskal–Wallis with Mann–Whitney *post hoc* test). Control (ctrl) stain was performed using the secondary antibody only. Goldner–Masson trichrome (GT) staining was used to visualize fractured site in mineralized bone cortex (green) (size bar: 100 µm). Selected areas are presented in higher magnification to better visualize PTX^+^ cells (indicated by arrowheads). **(B)** Representative microphotographs of the fractured areas with intensive endochondral ossification at day 6 postfracture. In the selected area, numerous hypertrophic PTX3^+^ chondrocytes are present during fracture healing.

Pentraxin 3 has been implicated in a number of inflammatory conditions (33); therefore, we further examined the time course of PTX3 expression during the early inflammatory phase of fracture healing in *ptx3^+/+^* mice. Immunohistochemistry revealed weakly positive osteocytes incorporated in the cortical bone matrix even in the unfractured bone tissue (Figure 5A). Moreover, expression of PTX3 is induced in the soft callus tissue early after the bone fracture injury, as evidenced by the significant increase of immunopositive area at day 6 postfracture (median, 5.3; IQR, 3.6–9.4 vs 0.4, IQR, 0.2–0.6 in control; *p* = 0.0012) (Figure 5A). Specifically, in areas of endochondral ossification, hypertrophying chondrocytes highly express PTX3 (Figure 5B). In parallel, serum level of PTX3 was increased at day 2 postfracture compared to unfractured *ptx3^+/+^* mice (Figure S5A in Supplementary Material).

Furthermore, we tested whether mice lacking the inflammatory mediator PTX3 show alterations in systemic and local fracture-induced inflammatory response at early postfracture days. Serum levels of the inflammatory chemokine CCL2 were comparable between *ptx3^+/+^* and *ptx3^−/−^* mice, with the peak value at day 2 postfracture (Figure S5A in Supplementary Material). Levels of TNF-α and IL-6 were undetectable in serum of both *ptx3^+/+^* and *ptx3^−/−^* mice (data not shown). Analysis of periosteal/callus tissue associated with fracture site revealed that an increase in CCL2 expression and accumulation of myeloid cell subpopulations (Ly6C^+^, Ly6G^+^) was associated with fracture injury (Figures S5B,C in Supplementary Material). At the selected time points of 2 and 6 days postfracture, neither TNF-α nor IL-1α showed significant changes at the gene expression level (Figure S5B in Supplementary Material), possibly because their increase occurs much earlier following injury. However, no obvious differences were observed in the expression of pro-inflammatory cytokines (CCL2 and IL-1α, Figure S5B in Supplementary Material; IL-6, not shown) or the frequency of inflammatory cell subsets (Ly6C^+^, Ly6G^+^, and CD11b^+^) between *ptx3^+/+^* and *ptx3^−/−^* mice (Figure S5C in Supplementary Material).

The fracture model used is based on the intramedullary insertion of a stainless steel pin that, *per se*, can induce an inflammatory response. Thus, we assessed in both *ptx3^+/+^* and *ptx3^−/−^* mice the contribution of injury made by the intramedullary pin insertion to the inflammatory response associated with the fracture model applied in this set of experiments. Expression of inflammatory mediators did not significantly differ between control (intact) animals and animals with inserted pins. Although pin insertion probably causes a certain level of intramedullary inflammatory response, bone injury and subsequent periosteal reaction seem to be necessary to produce increased expression of inflammatory markers in serum and callus tissue (Figure S5 in Supplementary Material).

**Figure 6:**
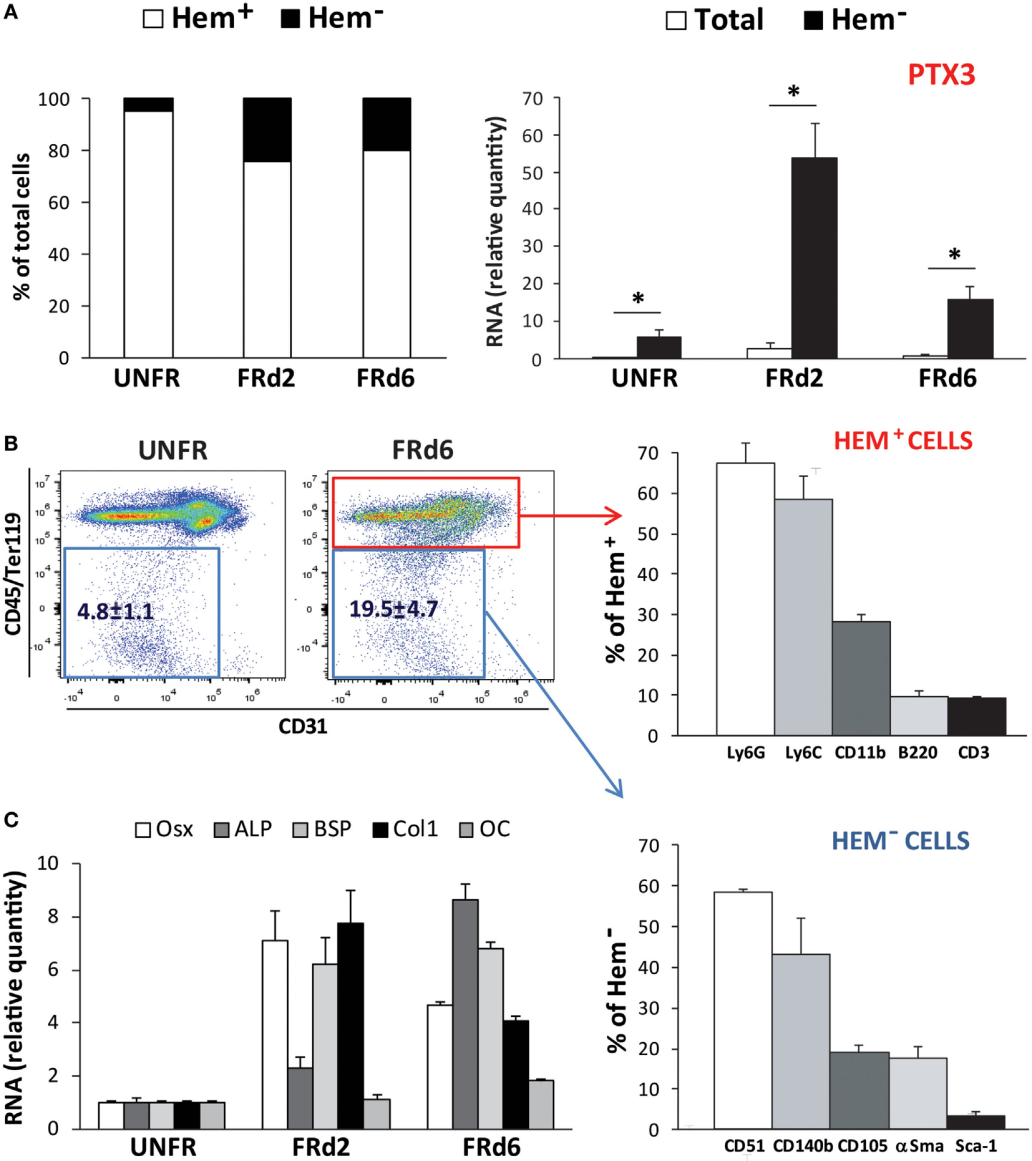
Callus composition at early postfracture days in *ptx3^+/+^* female mice. Stabilized closed transversal mid-tibial fracture model was applied in female *ptx3^+/+^* mice (14–16 weeks of age). Assessment of callus tissue at 2 (FRd2) and 6 (FRd6) days postfracture was performed by flow cytometry and qPCR. Flow cytometric analysis and cell sorting of hematopoietic (CD45^+^Ter119^+^CD31^+^) and non-hematopoietic/non-endothelial (CD45*^−^*Ter119*^−^*CD31*^−^*) subsets of callus cells were performed in unfractured tibial periosteal layer (UNFR) and fractured areas involving periosteal reaction (FRd2 and FRd6). **(A)** Percentage of hematopoietic (Hem^+^) and non-hematopoietic/non-endothelial (Hem*^−^*) cells in callus tissue (left panel). qPCR analysis of PTX3 gene expression in total callus cell population and non-hematopoietic/non-endothelial (Hem*^−^*) compartment of extracted cells (right panel). A total number of 12–16 mice per group were analyzed in 4 independent experiments. Values are shown as mean ± SD; statistically significant difference between corresponding total and Hem*^−^* groups is marked on plots (*p < 0.001; unpaired Student’s *t*-test). **(B)** Hematopoietic compartment (CD45^+^Ter119^+^CD31^+^ subset) and non-hematopoietic/non-endothelial compartment (CD45*^−^*Ter119*^−^*CD31*^−^* subset) were separately analyzed for the expression of hematopoietic lineage markers (Ly6G, Ly6C, CD11b, B220, CD3) and osteoprogenitor cell markers (CD51, CD140b, CD105, αSma, Sca-1), respectively. **(C)** Expression of osteoblast-specific genes (Osx, ALP, BSP, Col1, OC) were assessed at early postfracture days in total callus tissue by qPCR and expressed as relative quantity compared to periosteal samples of unfractured bones (*n* = 4 per group). ALP, alkaline phosphatase; BSP, bone sialoprotein; Col1, α2-chain of type I collagen; OC, osteocalcin; Osx, osterix; PTX3, pentraxin 3; αSma, α-smooth muscle actin.

Flow cytometry analysis of the callus cellular components showed that the percentage of non-hematopoietic/non-endothelial cells extracted from the callus tissue increased at early times from fracture (day 2 and 6). Expression of the PTX3 gene was strongly and selectively induced in non-hematopoietic/non-endothelial CD45*^−^*Ter119*^−^*CD31*^−^* population (Figure 6A). The presence of active osteoprogenitor cells in the callus was confirmed by high levels of expression of several bone-specific genes (Osx, ALP, BSP, and Col1). Non-hematopoietic subset accounted for ~20% of the callus cell population and expressed osteoprogenitor markers, including CD51, CD140b, and, to a lesser extent, CD105 and αSma (Figures 6B,C). The hematopoietic subset (CD45^+^Ter119^+^CD31^+^) comprised multiple cell lineages, mostly myeloid cells (Ly6C^+^, Ly6G^+^ and CD11b^+^) and some lymphoid cells (CD3^+^ and B220^+^; NK1.1^+^, data not shown). Nevertheless, the expression of PTX3 appears to be confined to the non-hematopoietic compartment, as no PTX3 mRNA could be detected in the hematopoietic subset at the analyzed time points (data not shown).

### PTX3 Expression Increases with Bone Cell Differentiation *In Vitro*

**Figure 7:**
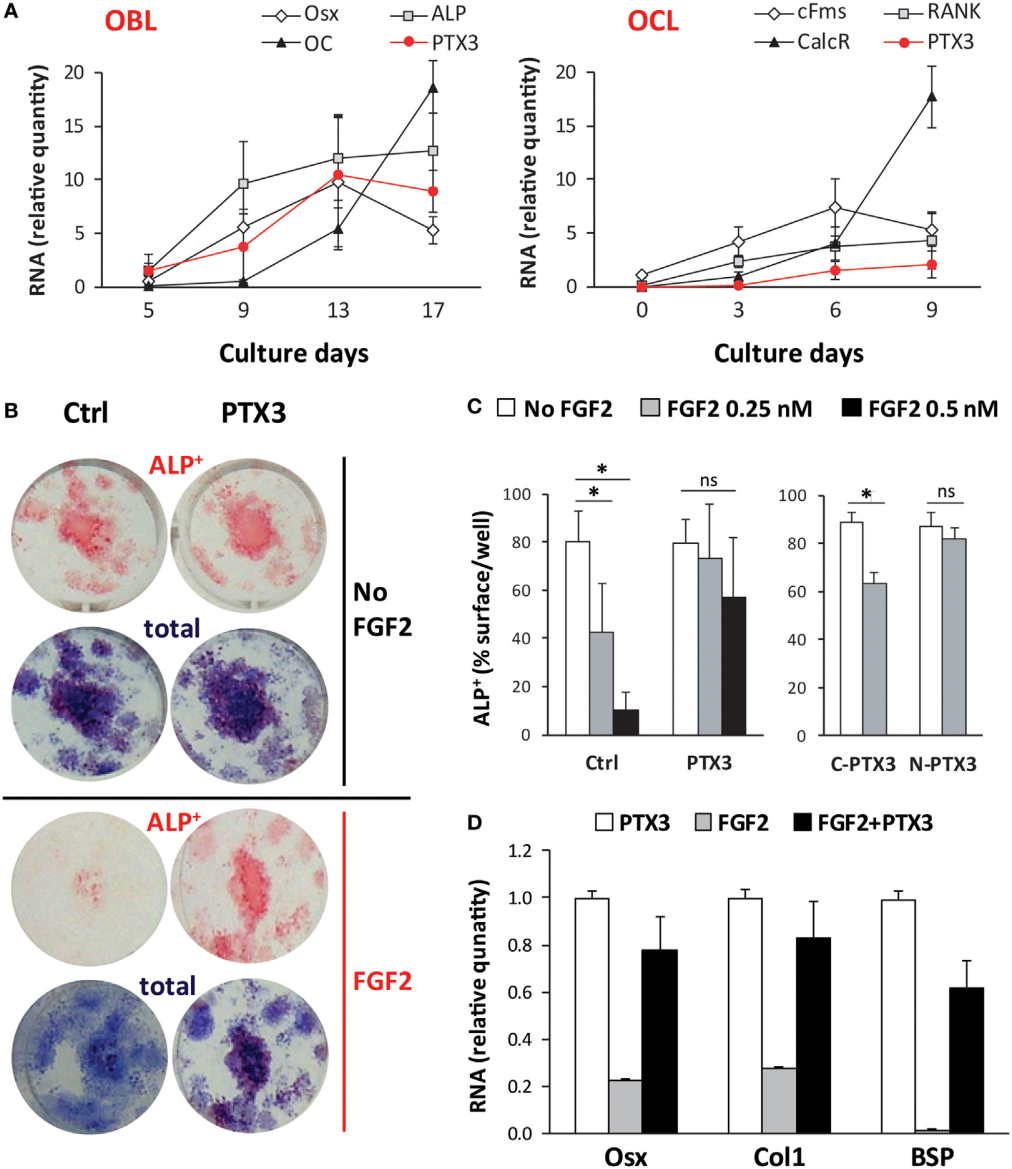
Role of PTX3 and FGF2 during *in vitro* differentiation of bone marrow cells from *ptx3^+/+^* female mice. Bone marrow cells were extracted from *ptx3^+/+^* female mice (10–12 weeks of age) and plated in OCL cultures (in the presence of RANKL and M-CSF) or OBL cultures (in the presence of AA and b-GP). Cells from two to three mice were pooled and three to four wells were done for each time point. **(A)** Expression of osteoblast-specific (Osx, ALP, OC) and osteoclast-specific (cFms, RANK, CalcR) genes were assessed during bone cell differentiation in parallel with the expression of PTX3 by qPCR. Mean ± SD from four independent experiments is reported. **(B)** Representative microphotographs of osteoblasts differentiated *in vitro* from bone marrow of *ptx3^+/+^* female mice on B6 background, cytochemically stained for ALP. Osteoblast differentiation was evaluated by measurement of ALP^+^ surface area per well and expression of osteoblast-specific genes (Osx, Col1, BSP). Total colony surface was assessed by methylene blue staining. OBL cultures were treated with PTX3 (50 nM) or/and FGF2 (0.5 nM) in addition to AA and β-GP. **(C)** Osteoblast differentiation was evaluated by measurement of surface area covered by ALP^+^ osteoblast after treatment with either full-length PTX3, its *N*- or *C*-terminal domain (all used at 50 nM) in addition to FGF2 (0.25 nM or 0.5 nM). The experiments were repeated three times (*n* = 6–8 wells per group). Values are presented as mean ± SD; statistically significant difference between corresponding FGF2-treated groups is marked on plots (*p < 0.05; ANOVA with Student–Newman–Keuls *post hoc* test). **(D)** Expression of osteoblast-specific genes (Osx, Col1, BSP) in the presence of PTX3 (50 nM), FGF2 (0.25 nM), or a combination of PTX3 and FGF2. AA, ascorbic acid; ANOVA, analysis of variance; ALP, alkaline phosphatase; BSP, bone sialoprotein; CalcR, calcitonin receptor; cFms, colony-stimulating factor 1 receptor; Col1, α2-chain of type I collagen; OBL, osteoblast; OC, osteocalcin; OCL, osteoclast; Osx, osterix; PTX3, pentraxin 3; RANK, receptor activator of nuclear factor-κB.

Next, we asked whether PTX3 could act as an autocrine growth factor for bone cells and characterized the kinetics of PTX3 expression (along with that of selected differentiation-specific genes) in osteoclastogenic and osteoblastogenic cultures (Figure 7A). Osteoblast differentiation (as induced by AA and β-GP) was confirmed by a large increase in the expression of both early and late osteoblast-specific genes (Osx, ALP, OC). In these conditions, PTX3 expression was highly induced at later time points. Osteoclastogenic cultures derived from bone marrow cells and stimulated with RANKL and M-CSF showed an increased expression of differentiation genes (cFms, RANK, and CalcR). PTX3 was also expressed in the course of osteoclast maturation, however, at lower levels (6- to 10-fold less) than those observed in the osteoblastogenic cultures (PTX3 was normalized using the same relative standard curve to allow comparison of the two cultures).

Although we observed a marked increase in PTX3 expression in mature osteoblasts *in vitro* and could expect, from previous findings, that PTX3 may directly stimulate osteoblast maturation, exogenously added PTX3 did not change the percentage of surface covered by ALP^+^ osteoblast (Figure S6A in Supplementary Material). Similarly, addition of PTX3 into osteoclastogenic cultures did not alter the number of mature TRAP^+^ multinucleated osteoclasts. Therefore, we proposed that PTX3 action on bone cells may be indirect, through interaction with some other bone matrix regulatory protein.

### PTX3 Binds to FGF2 and Reverses Its Inhibitory Role in Osteoblast Differentiation

To elucidate the mechanisms by which PTX3 is required for physiological bone formation and bone tissue regeneration after injury, we performed combined treatment of osteoblastogenic cultures with PTX3 and FGF2, a known regulator of osteoblastogenesis and also a ligand of PTX3 (34). As shown in Figures 7B,C, addition of FGF2 markedly reduced the percentage of surface covered by ALP^+^ osteoblast after culture treatment in a dose-dependent manner. This effect is only seen if FGF2 is added during the differentiation phase (day 7–14) of osteoblastogenic cultures and not during the proliferative phase (day 0–7), as described in Materials and Methods (Figure S6B in Supplementary Material). Addition of PTX3 to FGF2 treatment was able to reverse the negative effect of FGF2 on osteoblast differentiation (Figures 7B,C). The *N*-terminal domain of PTX3 [that contains the FGF2-binding site (35)] recapitulates the FGF2-neutralizing activity of the full lengths protein, whereas no effect was observed with the *C*-terminal domain (that does not interact with FGF2). In addition, FGF2 strongly suppressed the expression of the differentiation markers Osx, Col1, and BSP, whereas PTX3 rescued their expression toward control levels (Figure 7D).

**Figure 8:**
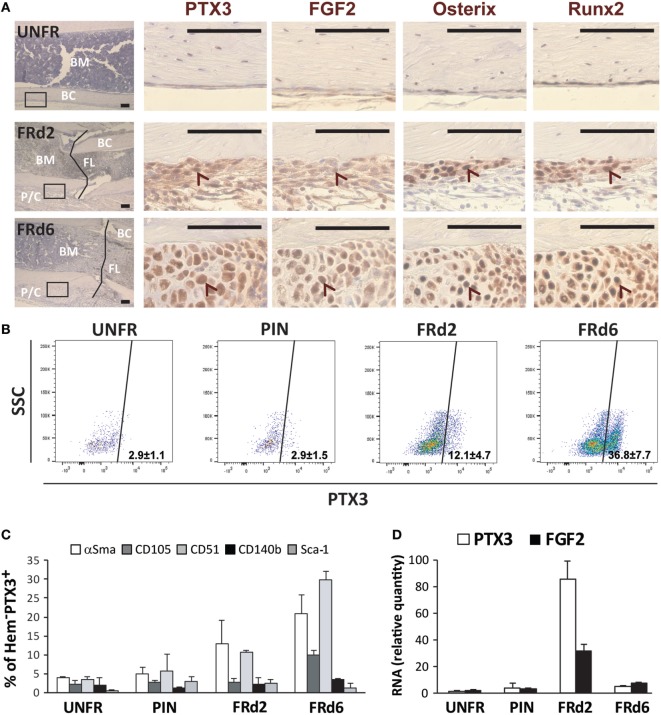
Dissection of osteoprogenitor subpopulations within the fractured areas at early postfracture days in *ptx3^+/+^* female mice. A stabilized closed transversal mid-tibial fracture model was applied in female *ptx3^+/+^* mice (14–16 weeks of age). Callus tissue was assessed at 2 (FRd2) and 6 (FRd6) days postfracture by immunohistochemistry, flow cytometry, and qPCR. **(A)** Expression of PTX3, FGF2, osterix, and Runx2 was analyzed by immunohistochemistry on serial sections in unfractured tibial periosteal layer (UNFR) and mid-tibial fracture areas (FRd2 and FRd6) from *ptx3^+/+^* female mice. Control stain (not shown) was performed using the secondary antibody only (size bar: 100 µm). Low-magnification images (far left panel, hematoxylin stained) is presented to show specimen orientation—intramedullary compartment comprising bone marrow (BM), bone cortex (BC), and, when applicable, fracture line (FL) and periosteal layer with callus tissue (P/C). Rectangles indicate area of interest shown at high magnification (size bar: 100 µm). Arrowheads indicate corresponding (positive) area on serial sections. **(B)** Flow cytometric analysis of PTX3^+^-positive subset within non-hematopoietic/non-endothelial (CD45*^−^*Ter119*^−^*CD31*^−^*) population of cells in unfractured tibial periosteal layer (UNFR), periosteal layer of bones with inserted pin (PIN), and fractured areas involving periosteal reaction and callus tissue (FRd2 and FRd6). **(C)** Flow cytometric analysis of osteoprogenitor subsets expressing αSma, CD105, CD51, CD140b, or Sca-1 marker within PTX3^+^ population [shown in **(B)**] for female *ptx3^+/+^* mice (*n* = 6–8 mice per group). **(D)** qPCR analysis of PTX3 and FGF2 gene expression in non-hematopoietic/non-endothelial (CD45*^−^*Ter119*^−^*CD31*^−^*) compartment of cells extracted from unfractured tibial periosteal layer (UNFR), periosteal layer of bones with inserted pin (PIN), and mid-tibial fracture areas (FRd2 and FRd6) of female ptx3^+/+^ mice (*n* = 3–4 per group).

Finally, FGF2 was detected (both protein and mRNA) at early days from fracture and showed a similar pattern of expression as PTX3 in bone sections and extracted callus cells of *ptx3^+/+^* mice (Figures 8A,D). Moreover, immunohistochemical staining of serial bone sections indicated concomitant high expression of Osx and Runx2 by infiltrating osteoprogenitor cells in the areas of periosteal reaction and bone induction (Figure 8A). The cellular source of PTX3 was determined by intracellular staining of PTX3 in parallel to the staining of surface hematopoietic (data not shown) and mesenchymal/osteoprogenitor markers using flow cytometry (Figures 8B,C). The percentage of PTX3^+^ cells increased following fracture (Figure 8B), and among them, the most abundant subsets were αSma^+^ early osteoprogenitors and CD51^+^ more mature preosteoblast (Figure 8C), whereas hematopoietic cells exhibited almost no PTX3 expression at day 2 and 6 postfracture (data not shown). There were no differences in PTX3 expression between control (intact) animals and animals with inserted pins (Figure 8B). A similar pattern of expression for FGF2, Osx, and Runx2 was observed in bone sections of *ptx3^−/−^* mice, with no PTX3 immunoreactivity (Figure S7 in Supplementary Material). In addition, the percentages of hematopoietic/endothelial-negative (CD45*^−^*Ter119*^−^*CD31*^−^*) osteoprogenitor subpopulations (CD51^+^ and CD140b^+^, Figure S8 in Supplementary Material; CD105, data not shown) were comparable between *ptx3^+/+^* and *ptx3^−/−^* mice except for the αSma^+^ subset that was lower in *ptx3^−/−^* mice (Figure S8 in Supplementary Material).

On the basis of these findings we propose that PTX3, produced by osteoblast lineage cells, serves as a bone protective factor, important to unlock osteoblast maturation by neutralizing the FGF2 anti-differentiation effect during bone formation.

## Discussion

In this study, we identified a new role for the long pentraxin PTX3 as a local regulator of bone metabolism. We observed that young adult *ptx3^−/−^*mice (2.5 months of age) have lower trabecular bone mass in long bones and axial skeleton, indicating that genetic deficiency of PTX3 alters the balance between bone formation and resorption in both B6 and S129 strain. Skeletal sexual dimorphism is present in *ptx3^−/−^* mice, since more obvious bone phenotype compared to *ptx3^+/+^* mice has been observed for young adult females, known to have lower BFR compared to male mice at the analyzed age (36). Levels of several hormones, cytokines, and growth factors important for osteoblast function may be different in females vs males (including sex hormones, growth hormone, and RANKL), making them susceptible to specific conditions, i.e., PTX3 deficiency (37). Compared to 2.5 months, the bone phenotype was less pronounced in females at 6–8 months when age-related decline in osteoblast functions and trabecular bone loss have already begun (38, 39). Adult bone is a dynamic tissue that requires constant remodeling to maintain structural integrity and fulfill its metabolic roles. In addition to hormonal signals and mechanical stimuli, paracrine effects of local mediators, including tissue growth factors, cytokines, and bone matrix proteins are important for physiological and pathological bone turnover (15). The trabecular bone is particularly susceptible to modulatory signals and may undergo a negative bone balance in the adult organism due to high metabolic rate and great surface area-to-volume ratio (21, 38, 40, 41). The presence of lower bone mass in the spine of *ptx3^−/−^* mice, which in small quadrupeds is loaded by much lower forces compared to the femur, suggests that PTX3 is required for bone homeostasis not only in weight bearing, rapidly remodeled bones, but also in the axial skeleton.

Bone matrix incorporates a large number of growth factors, including insulin-like growth factors, transforming growth factor-β, BMPs, platelet-derived growth factor, and parathyroid hormone-related protein and FGFs, which play a role in the physiologic bone development and in the remodeling associated with skeletal repair. Matrix proteins mostly produced by the bone cells themselves, such as Col1, ALP, osteonectin, fibronectin, and OC, may act as growth factor modulators (16). They participate in the regulation of proliferation, migration, and differentiation of osteoblastic cells, especially during the resorption phase of bone remodeling when osteoclasts degrade bone matrix, releasing the incorporated proteins. Moreover, a number of inflammatory mediators including cytokines, chemokines, prostaglandins, and leukotrienes are produced by bone and immune cells and, through autocrine and paracrine loops, regulate bone cell functions (13, 22). However, these inflammatory mediators have been mostly studied in the context of pathological inflammation (i.e., arthritis, periodontitis, osteomyelitis), whereas our study determined the role of PTX3 in physiological bone remodeling and repair. We found that PTX3 is expressed by differentiating osteoblasts *in vitro* and matrix-embedded osteocytes *in vivo* and that PTX3-deficient mice have significantly lower bone formation and bone repair rate than *ptx3^+/+^* controls. Nevertheless, frequency and differentiation potential of bone marrow-derived osteoprogenitors *in vitro* were similar in *ptx3^+/+^* and *ptx3^−/−^* mice, indicating that an *in situ* microenvironment is required for the bone-spearing effect of PTX3. In addition, culture treatment with exogenous PTX3 did not directly affect osteoclast or osteoblast differentiation, similar to what observed by Lee et al. (42). This study also showed that PTX3 enhanced the RANKL expression in primary mouse calvarial osteoblasts and stimulated osteoclastogenesis under high TNF-α conditions. Other authors have investigated the role of PTX3 in chronic inflammatory diseases with aggravated bone resorption, including periodontitis (43–45), arthritis (46–52), and tumor-associated inflammation (53, 54), showing increased PTX3 levels, especially in areas of inflammation. However, in these models, it is difficult, if not impossible, to define whether PTX3 is the causal factor of bone loss or it is just a byproduct, downstream of pro-inflammatory cytokines that *per se* induce bone resorption.

As opposed to chronic inflammatory diseases or excessive inflammation associated with massive trauma, when the inflammatory response has destructive effects on bone tissue, inflammation plays an immediate beneficial role in promoting regeneration after bone fracture or injury (19, 20). A number of inflammatory cytokines (TNF-α, IL-1α, IL-1β, and IL-6) are expressed during the acute inflammatory phase within a week from fracture, which, in cooperation with growth factors and matrix proteins released from injured tissues, recruit inflammatory cells, promote angiogenesis, and guide mesenchymal progenitor cell migration and differentiation (19). In our fracture model, the pro-inflammatory chemokine CCL2 was particularly useful as an indicator of early fracture injury in serum and at the fracture site. Later on, remodeling of the mineralized callus by the resorptive action of osteoclasts is paralleled by a second peak of inflammatory cytokines and matrix proteins. Although the recruited inflammatory cells, mainly neutrophils and macrophages, are major immediate producers of cytokines and growth factors, in the regenerative callus, these mediators are primarily produced by bone cells, mainly osteoblasts and chondrocytes (55). Here, we showed that PTX3 is expressed in both the early (inflammatory) and the late (regenerative) phases of fracture healing. Moreover, it is highly induced in non-hematopoietic/non-endothelial population of early callus tissue, which is mainly composed of osteoprogenitor cells expressing mesenchymal/osteoblast lineage markers (i.e., αSma, CD51, CD140b). In particular, PTX3 is produced by a substantial proportion of CD51^+^ and αSma^+^ subsets of osteoprogenitor cells. Moreover, the proportion of αSma^+^ subset, which label osteoprogenitor cells induced at early postfracture days (26), was significantly decreased in *ptx3^−/−^* mice. In a model of wound healing, Doni et al. also showed that PTX3 was mainly produced by non-hematopoietic mesenchymal lineage cells (9). Balanced cytokine *milieu* is crucial for the beneficial effect of acute inflammation on bone regeneration, so alterations in the local concentration of growth factors and inflammatory mediators may have significant impacts on the healing process. In mice lacking PTX3, callus mineralization is delayed with lower expression of Col1, the most abundant osteoblast-specific matrix protein. Despite lacking PTX3, the overall profile of systemic and fracture-associated inflammatory indicators (CCL2, IL-1α, IL-6) and inflammatory cell subsets (Ly6G, Ly6C, CD11b) in *ptx3^−/−^* mice showed no significant difference compared to *ptx3^+/+^* mice. To the best of our knowledge, there are no other studies investigating the role of PTX3 during bone regeneration. However, in the model of orthodontic tooth movement, characterized by a rapid bone remodeling, PTX3, together with other inflammatory mediators, was significantly overexpressed throughout the periodontal ligament of the tension zone (56). Other *in vivo* studies have demonstrated that mice lacking pro-inflammatory cytokines, such as IL-6 or TNF-α, or treated with anti-inflammatory drugs, such as cyclooxygenase-2 inhibitors, have disturbed bone formation and delayed fracture healing (19, 20, 22). Osteoblasts taken from human patients with fracture non-unions have abnormal expression of genes associated with inflammatory response, suggesting that inflammatory mediators are critical for bone healing (57).

Bone remodeling and regeneration relies on tight spatiotemporal interactions between growth factors and extracellular matrix molecules, which, in an autocrine and paracrine manner, promote osteoblast development and differentiation. Our results and previous studies showed that human and animal stromal/osteoblast lineage cells express PTX3 (9, 26, 58, 59). Moreover, a recent study by Scimeca et al. indicated importance of PTX3 for human osteoblast differentiation and showed reduced PTX3 expression in osteoblasts from osteoporotic patients (60). The multifunctional properties of PTX3 are based on its capacity to interact with several ligands, including complement components, adhesion molecules, growth factors, and matrix components (9, 25). In particular, PTX3 binds with high affinity to FGF2 (34, 35) and to other members of the FGF family (28, 61) *via* the *N*-terminal domain and prevents FGF2 from interacting with its receptor. The FGF family comprises 22 members, highly conserved in evolution (62), exerting various biological activities both *in vivo* and *in vitro*, including roles in angiogenesis, mitogenesis, cellular differentiation, cell migration, and tissue injury repair. In addition, FGF2 is expressed during the early stages of bone formation and is abundantly accumulated in bone matrix, participating to osteoblastogenesis and skeletal remodeling (63–65). Therefore, we propose that PTX3 might exert a bone regulatory effect in repair and remodeling through the interaction with locally produced FGF2. At early stages following fracture, non-hematopoietic/non-endothelial cells comprising the osteoprogenitor population showed concomitant induction in PTX3 and FGF2 gene expression. The FGF2 protein was detected by immunohistochemistry in close proximity to the fracture gap, overlapping with the areas of PTX3 staining and osteoprogenitor cells expressing Osx and Runx2. FGF2 is a key mitogen able to expand the pool of various bone cells, including osteoprogenitors. However, its effects in the later stages of matrix maturation and mineralization are controversial, with both positive and negative impacts described (66, 67). Genetic studies in animals and etiology of human skeletal dysplasias showed that FGF2 overexpression as well as mutation of FGF2 or the corresponding receptors cause bone abnormalities (65, 68). Similar to that described by Kalajzic et al. (69), in our *in vitro* conditions, FGF2 blocked osteoblast differentiation from bone marrow stromal cells, whereas PTX3 (and the PTX3 *N*-terminal domain that contains the FGF2-binding sites) was able to reverse the inhibitory effect of FGF2. Similarly, by sequestering FGF2, PTX3 can inhibit FGF2-dependent endothelial cell proliferation *in vitro* and angiogenesis *in vivo* (33, 35). Several extracellular matrix molecules, besides PTX3, are able to trap FGF2 in the extracellular environment (such as thrombospondin-1 and cleaved syndecan) and modulate its effects during processes such as inflammation, wound healing, atherosclerosis, and neoplasia (70, 71).

In conclusion, we observed that PTX3*-*deficient mice have impaired bone formation during physiological remodeling as well as regeneration following fracture injury. In particular, PTX3 is important for adequate osteoblast differentiation and proper matrix mineralization, and this effect is achieved, at least in part, by sequestering FGF2 in the extracellular matrix of newly remodeled bone. We propose that the role of PTX3 in the regulation of bone remodeling is a part of its general biological function in the orchestration of tissue repair.

## Author Contributions

DG, IK, AM, and BB designed the study; DG, LD, HC, VK, and TK performed the experiments; DG, MS, SV, LD, HC, NK, VK, and TK acquired and analyzed data; DG, MS, SV, AI, NK, IK, AM, and BB interpreted the results; DG, AI, AM, and BB prepared the manuscript. All authors critically revised the manuscript and approved the final version.

## Conflict of Interest Statement

The authors declare that the research was conducted in the absence of any commercial or financial relationships that could be construed as a potential conflict of interest.
