# Supplementary material for: The Long Pentraxin 3 Plays a Role in Bone Turnover and Repair
Source: Front Immunol. 2018 Mar 5;9:417. doi: 10.3389/fimmu.2018.00417 (PMC5845433; doi:10.3389/fimmu.2018.00417)
Supplement: Supplementary file 1 [file Data_Sheet_1.PDF]

Supplementary material

The long pentraxin PTX3 plays a role in bone turnover and repair

Grčević Danka, Sironi Marina, Valentino Sonia, Deban Livija, Cvija Hrvoje, Inforzato Antonio, Kovačić Nataša, Katavić Vedran, Kelava Tomislav, Kalajzić, Ivo, Mantovani Alberto, Bottazzi Barbara*

* **Corresponding Author:**
Barbara Bottazzi
Barbara.Bottazzi@humanitasresearch.it

Supplementary figures

Figure S1

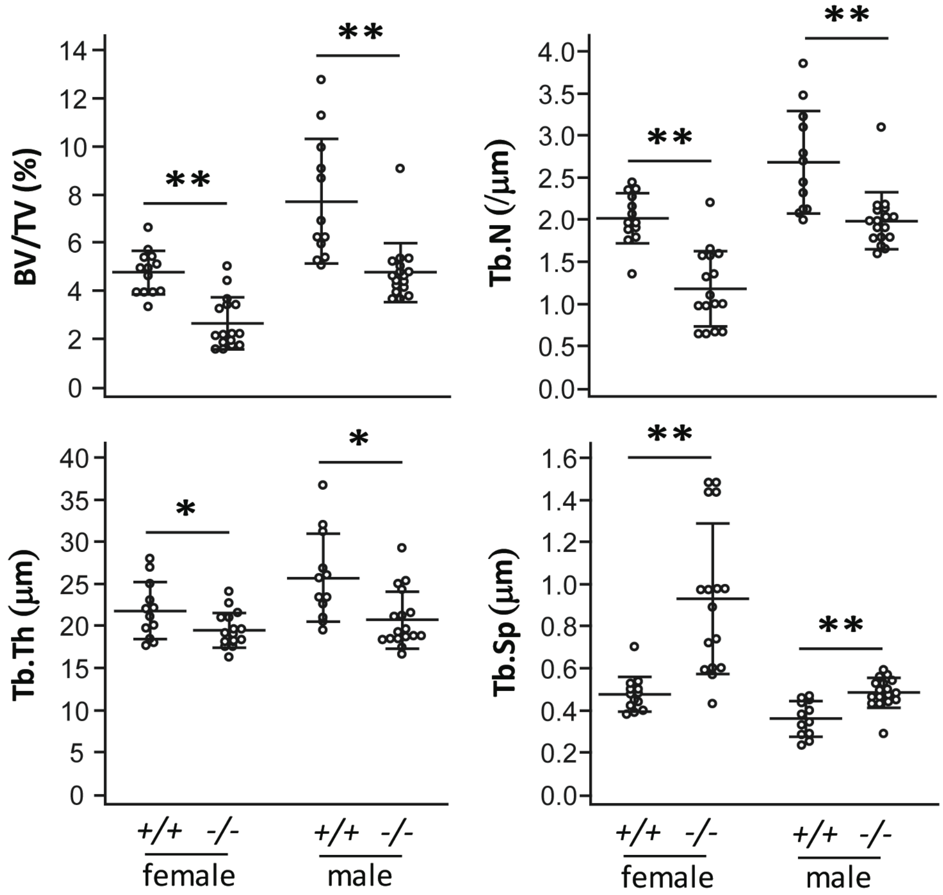

Figure S1: Bone phenotype of appendicular skeleton in young adult *ptx3*$^{+/+}$and *ptx3*$^{-/-}$ mice on B6 background assessed by histomorphometry. Female and male *ptx3*$^{+/+}$and *ptx3*$^{-/-}$ mice (10-12 weeks of age) were sacrificed, and the distal femora were analyzed for trabecular bone parameters: trabecular bone volume (BV/TV, bone volume/total volume), trabecular number (Tb.N), trabecular thickness (Tb.Th) and trabecular separation (Tb.S). Cumulative data from 2 independent sets of experiments are shown (n=12 to 16 mice per group). Dots represent individual mice, horizontal lines and error bars are mean ± standard deviation; statistically significant difference between corresponding *ptx3*$^{+/+}$and *ptx3*$^{-/-}$ groups is marked on plots (* $p<0.05$, ** $p<0.001$; unpaired Student's *t*-test).

Figure S2

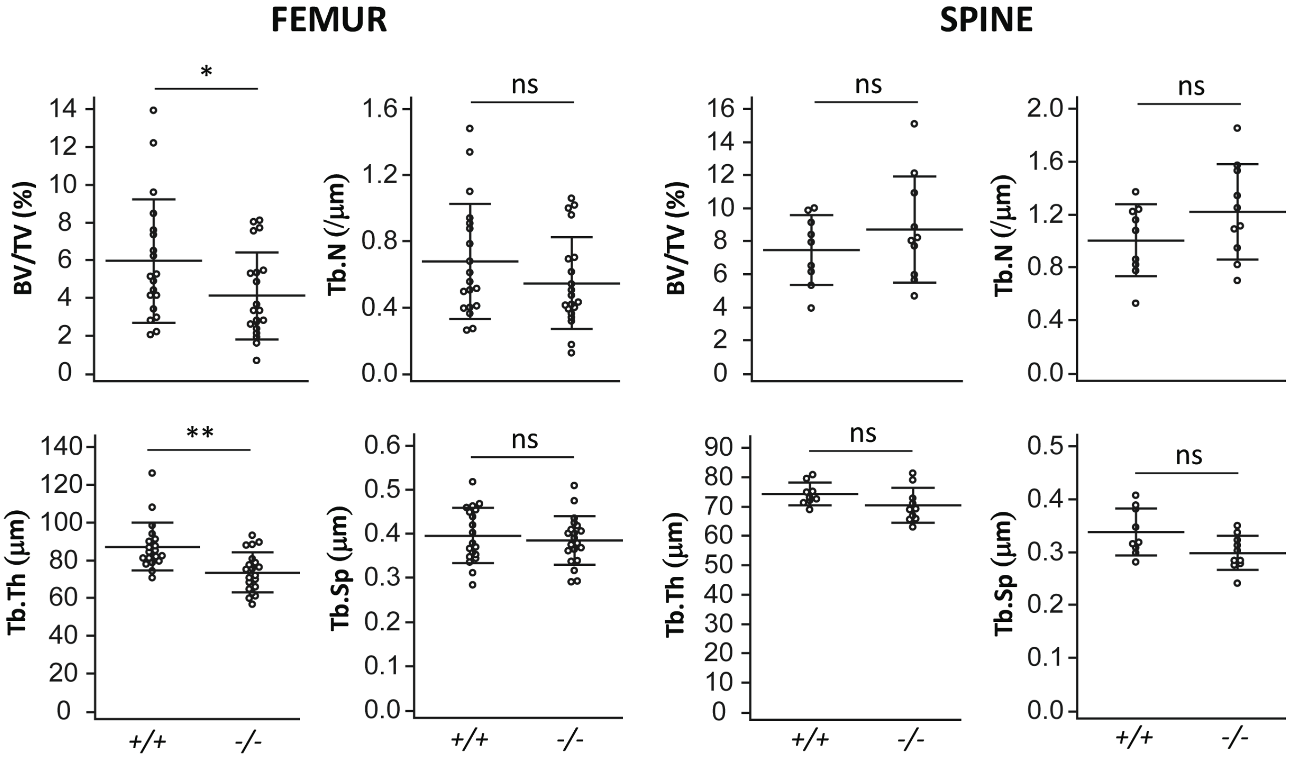

Figure S2. Bone phenotype of axial and appendicular skeleton in aged *ptx3*$^{+/+}$and *ptx3*$^{-/-}$ mice on B6 background assessed by µCT. Female *ptx3*$^{+/+}$and *ptx3*$^{-/-}$ (6-8 months of age) were sacrificed, and the distal femoral metaphysis (left panel) and second lumbar vertebra (right panel) were analyzed for trabecular bone parameters: trabecular bone volume (BV/TV, bone volume/total volume), trabecular number (Tb.N), trabecular thickness (Tb.Th) and trabecular separation (Tb.Sp). Cumulative data from 2 independent sets of experiments are shown (n=9-19 mice per group). Dots represent individual mice, horizontal lines and error bars are mean ± standard deviation; statistically significant difference between corresponding *ptx3*$^{+/+}$and *ptx3*$^{-/-}$ groups is marked on plots (* $p<0.05$, ** $p<0.001$; unpaired Student's *t*-test).

Figure S3

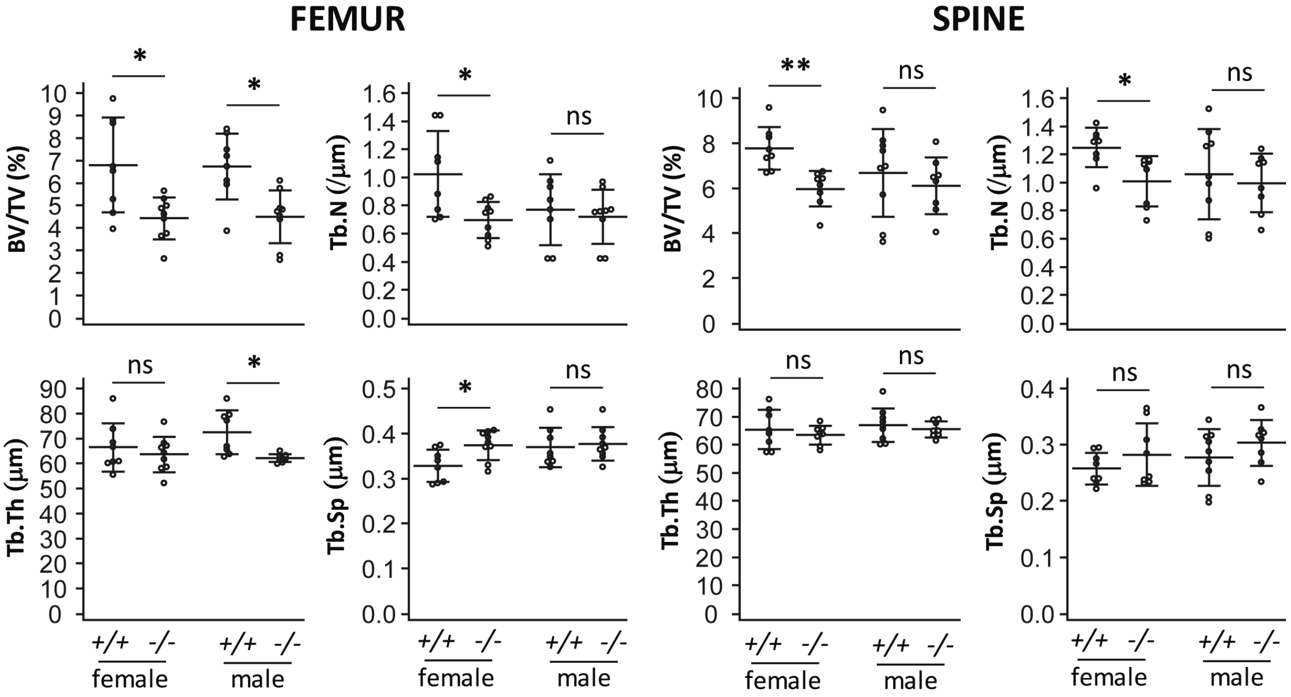

Figure S3. Bone phenotype of axial and appendicular skeleton in young adult *ptx3*$^{+/+}$and *ptx3*$^{-/-}$ mice on SV129 background assessed by µCT. Female and male *ptx3*$^{+/+}$and *ptx3*$^{-/-}$ mice (10-12 weeks of age) were sacrificed, and the distal femora (left panel) and second lumbar vertebra (right panel) were analyzed for trabecular bone parameters: trabecular bone volume (BV/TV, bone volume/total volume), trabecular number (Tb.N), trabecular thickness (Tb.Th) and trabecular separation (Tb.Sp). Cumulative data from 2 independent sets of experiments are shown (n=8 to 10 mice per group). Dots represent individual mice, horizontal lines and error bars are mean ± standard deviation; statistically significant difference between corresponding *ptx3*$^{+/+}$and *ptx3*$^{-/-}$ groups is marked on plots (* $p<0.05$, ** $p<0.001$; unpaired Student's *t*-test).

Figure S4

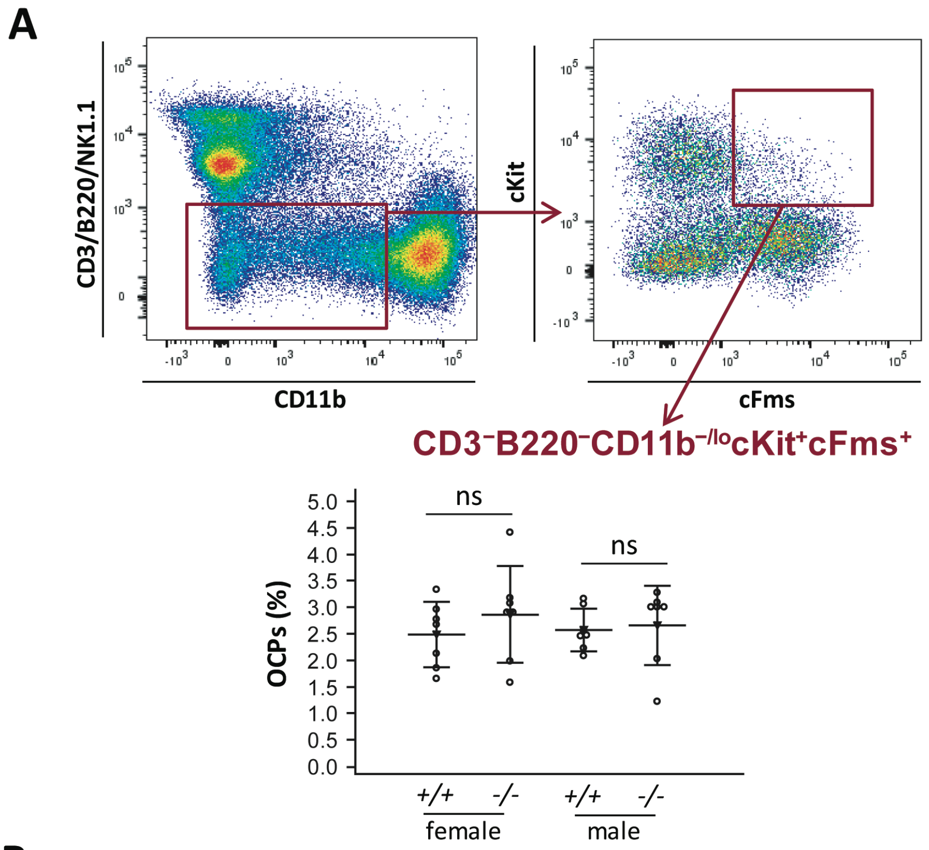
A
CD3/B220/NK1.1
CD11b
cKit
cFms
CD3⁻B220⁻CD11b⁻/lo cKit⁺cFms⁺
OCPs (%)
ns
ns
+/+
-/-
+/+
-/-
female
male

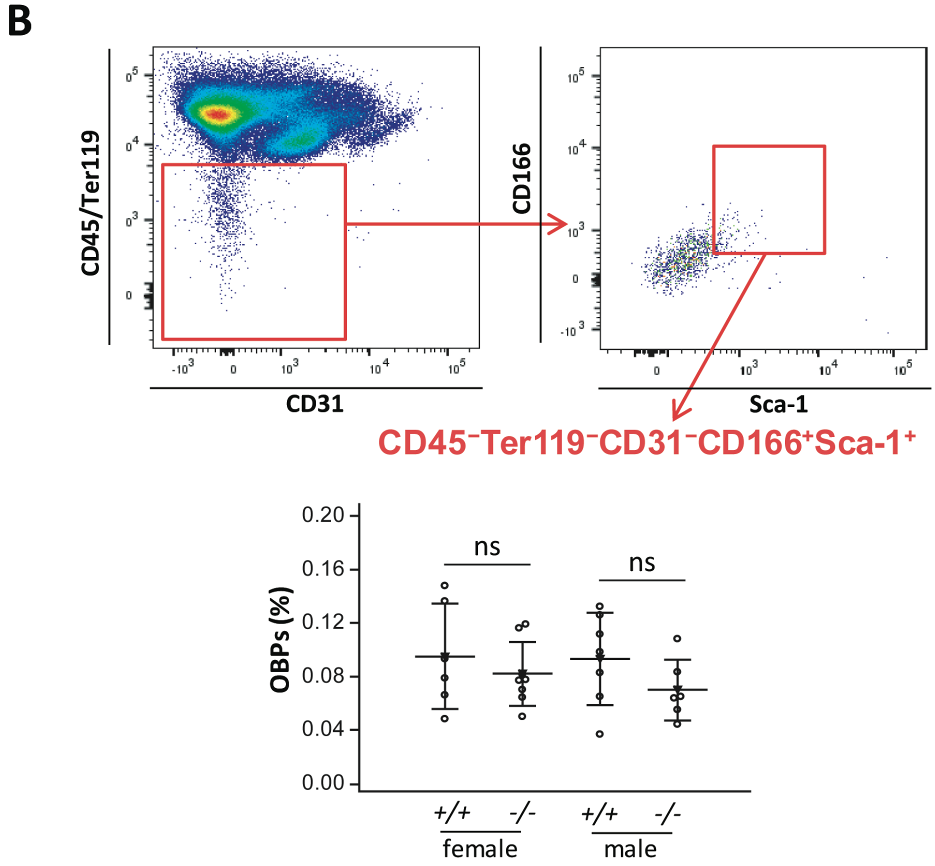
B
CD45/Ter119
CD31
CD166
Sca-1
CD45⁻Ter119⁻CD31⁻CD166⁺Sca-1⁺
OBPs (%)
ns
ns
+/+
-/-
+/+
-/-
female
male

64

Figure S4. Frequency of bone marrow osteoclast and osteoblast progenitor cells in *ptx3*$^{+/+}$ and *ptx3*$^{-/-}$ mice on B6 background. Bone marrow cells were extracted from female and male *ptx3*$^{+/+}$ and *ptx3*$^{-/-}$ mice (10-12 weeks of age) and prepared for flow-cytometric analysis. (A) Representative FACS dot-plots and quantitative assessment of the frequency of bone marrow osteoclast progenitor cells (OCPs) ($CD3^-B220^-CD11b^{-/low}CD115^+CD117^+$ cells) in *ptx3*$^{+/+}$ and *ptx3*$^{-/-}$ mice. (B) Representative FACS dot-plots and quantitative assessment of the frequency of osteoblast progenitors (OBPs) ($CD45^-Ter119^-CD31^-CD166^+Sca\text{-}1^+$ cells) in the bone marrow of *ptx3*$^{+/+}$ and *ptx3*$^{-/-}$ mice. Analysis was repeated in 3 independent sets of experiments (n=6 to 8 per group). Dots represent individual values, horizontal lines and error bars are mean ± standard deviation; no statistically significant difference was observed between corresponding *ptx3*$^{+/+}$ and *ptx3*$^{-/-}$ groups (unpaired Student's *t*-test).

Figure S5

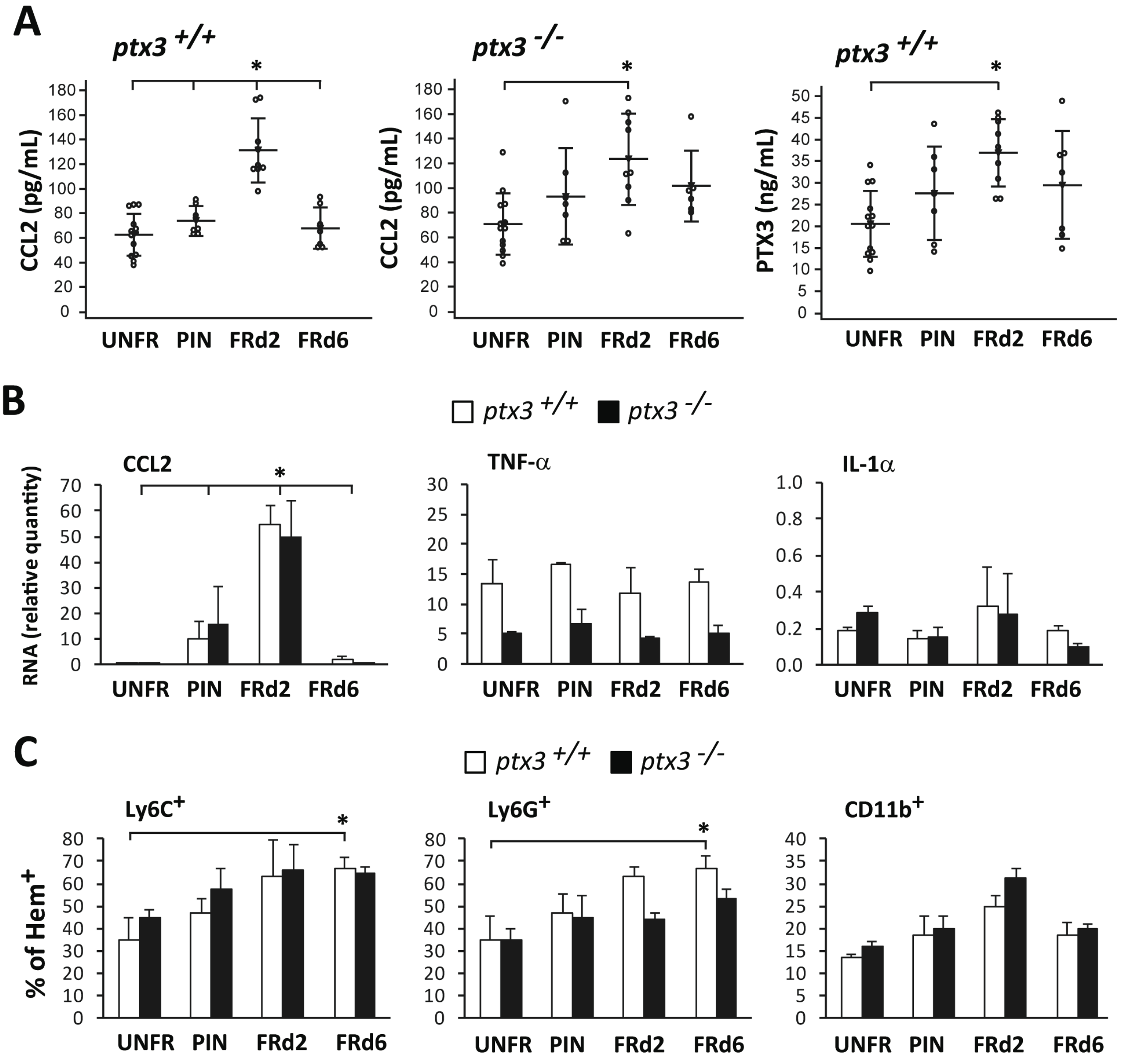

Figure S5. Inflammatory mediators and myeloid lineage distribution at early post-fracture days in *ptx3*$^{+/+}$and *ptx3*$^{-/-}$ mice on B6 background. A stabilized closed transversal mid-tibial fracture model was applied in female *ptx3*$^{+/+}$and *ptx3*$^{-/-}$ mice (14-16 weeks of age). Serum samples and callus tissue were harvested from unfractured mice (UNFR), mice with inserted pins (PIN) and mice at 2 (FRd2) and 6 (FRd6) days post-fracture, and analyzed by ELISA, qPCR and flow cytometry. (A) Concentrations of inflammatory mediators CCL2 and PTX3 in sera of *ptx3*$^{+/+}$and *ptx3*$^{-/-}$ mice at early post-fracture days measured by ELISA (n=7 to 13 mice per group). PTX3 was undetectable in *ptx3*$^{-/-}$ mice (data not shown). Dots represent individual mice, horizontal lines and error bars are mean ± standard deviation; statistically significant difference between time-points within the *ptx3*$^{+/+}$and *ptx3*$^{-/-}$ groups is marked on plots (* $p<0.05$; ANOVA and Student-Neuman-Keuls post-

hoc test). (B) Gene expression of inflammatory mediators in callus tissue of *ptx3*$^{+/+}$and *ptx3*$^{-/-}$ mice at early post-fracture days assessed by qPCR. Values are presented as mean ± standard deviation (n=6-8 mice per group); statistically significant difference between time-points within the *ptx3*$^{+/+}$ or *ptx3*$^{-/-}$ groups is marked on plots (* $p<0.05$; ANOVA and Student-Neuman-Keuls post-hoc test). (C) Distribution of inflammatory cell subsets (Ly6C, Ly6G, CD11b) within hematopoietic-positive population (Hem^{+}; CD45^{+}Ter119^{+}CD31^{+}) of cells in unfractured tibial periosteal layer (UNFR), periosteal layer of bones with inserted pin (PIN) and fractured areas involving periosteal reaction and callus tissue (FRd2 and FRd6) from *ptx3*$^{+/+}$and *ptx3*$^{-/-}$ mice. Values are presented as mean ± standard deviation (n=6-8 mice per group); statistically significant difference between time-points within the *ptx3*$^{+/+}$ groups is marked on plots (* $p<0.05$; ANOVA and Student-Neuman-Keuls post-hoc test).

Figure S6

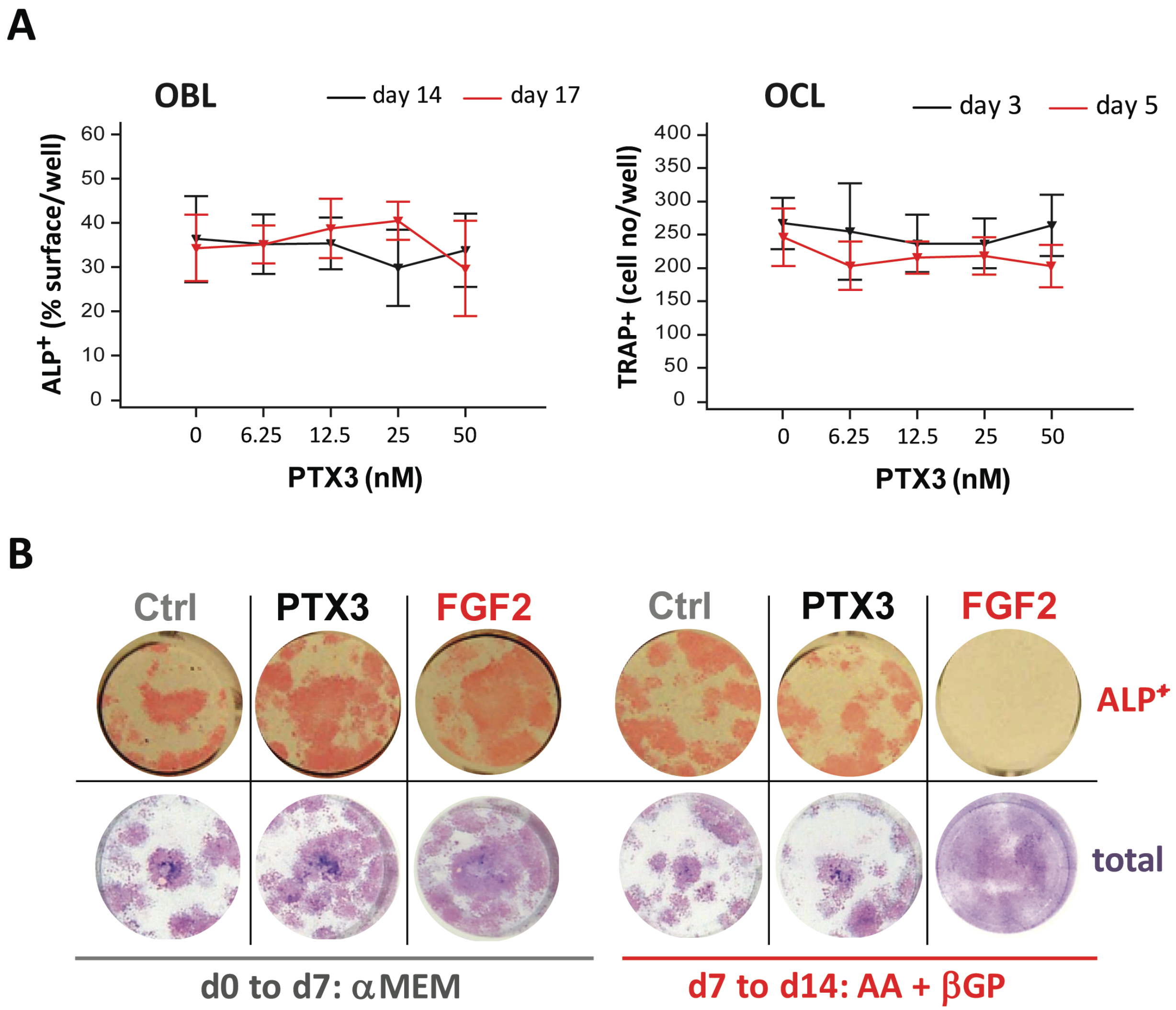

Figure S6. Effect of PTX3 and FGF2 treatment on bone cell differentiation in *ptx3*$^{+/+}$ female mice on B6 background. (A) Bone marrow cells were extracted from *ptx3*$^{+/+}$ female mice (10-12 weeks of age) and plated in osteoclastogenic cultures (by the addition of RANKL and M-CSF) or osteoblastogenic cultures (by the addition of AA and β-GP). Cells from 2 to 3 mice were pulled; 3 to 4 wells were done for each culture condition. A dose range of PTX3 (0 to 50 nM) was added to osteoclast and osteoblast cultures, and the effects on the number of TRAP^{+} osteoclasts and ALP^{+} percent surface were assessed respectively. The experiments were repeated two times (n=6 wells per group for osteoblasts; n=8 wells per group for osteoclasts). Values are presented as mean ± standard deviation. (B) Representative microphotographs of osteoblasts differentiated *in vitro* from bone marrow of *ptx3*$^{+/+}$ female mice. Osteoblastogenic cultures were grown for two weeks: during the first week bone-marrow derived fibroblast were cultured in aMEM/10% FCS to stimulate cell proliferation; during the second week, adherent cells were treated with AA and β-GP to stimulate osteoblast lineage differentiation. In addition, cultures were treated with PTX3 (50 nM) or FGF2

(0.5 nM). The experiments were repeated three times (n=6 to 8 wells per group). Osteoblast differentiation was evaluated by staining of ALP^+ osteoblast. Total colony area was assessed by methylene blue staining.

Figure S7

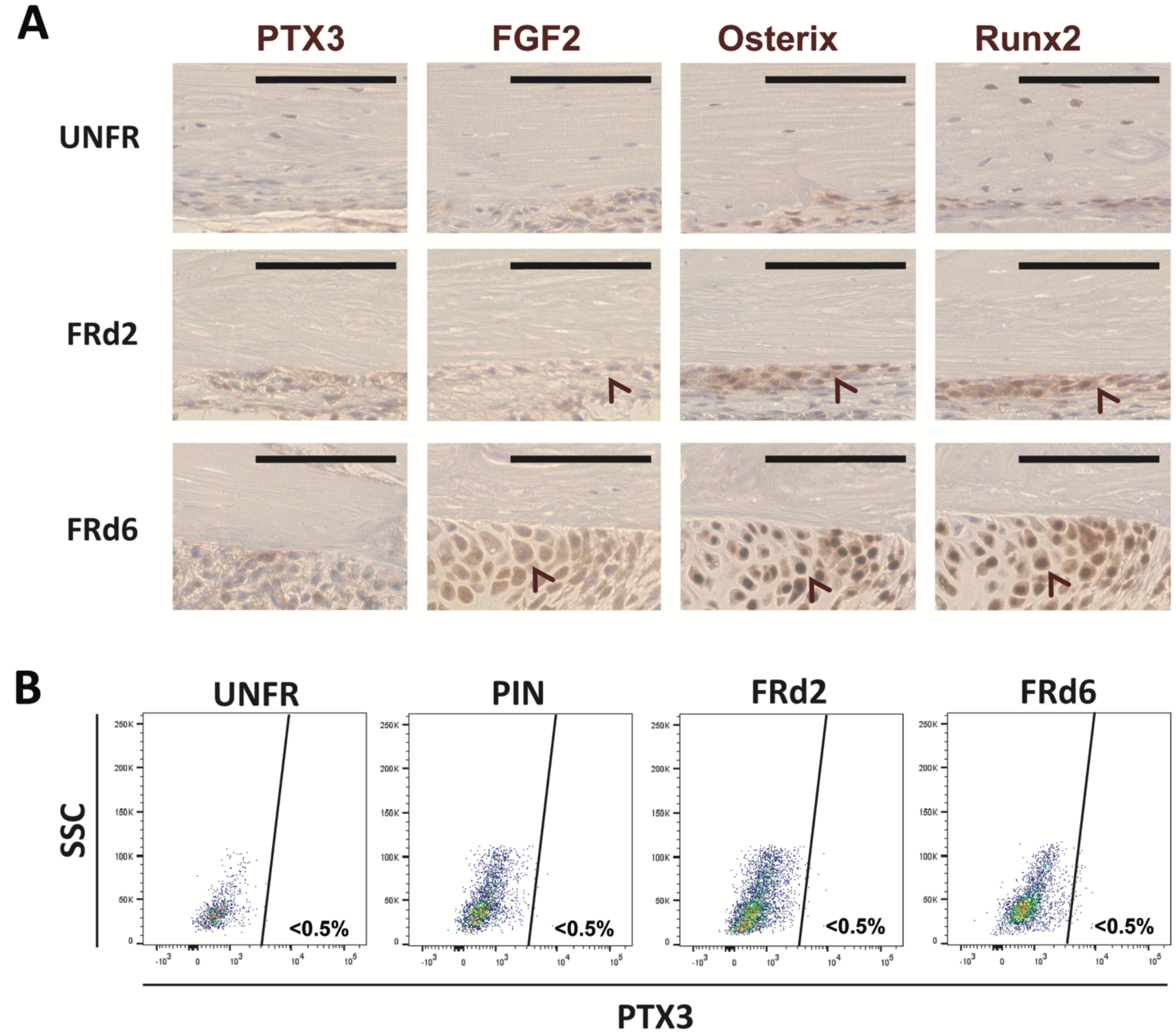

Figure S7. Dissection of osteoprogenitor subpopulations within the fractured areas at early post-fracture days in *ptx3*$^{-/-}$ female mice. A stabilized closed transversal mid-tibial fracture model was applied in female *ptx3*$^{-/-}$ mice (14-16 weeks of age). (A) Expression of PTX3, FGF2, osterix and Runx2 was analyzed by immunohistochemistry on serial sections in unfractured tibial periosteal layer (UNFR) and mid-tibial fracture areas (FRd2 and FRd6). Control stain (not shown) was performed using the secondary antibody only (size bar: 100 µm). Arrowheads indicate corresponding (positive) area on serial sections, not applicable for anti-PTX3 staining in *ptx3*$^{-/-}$ mice. (B) Specificity of anti-PTX3 labeling was confirmed by signal absence in *ptx3*$^{-/-}$ mice. Flow cytometric analysis of PTX3^{+} positive subset within non-hematopoietic/non-endothelial (CD45^{-} Ter119^{-}CD31^{-}) population of cells in unfractured tibial periosteal layer (UNFR), periosteal layer of bones with inserted pin (PIN) and fractured areas involving periosteal reaction and callus tissue (FRd2 and FRd6; n=6-8 mice per group).

Figure S8

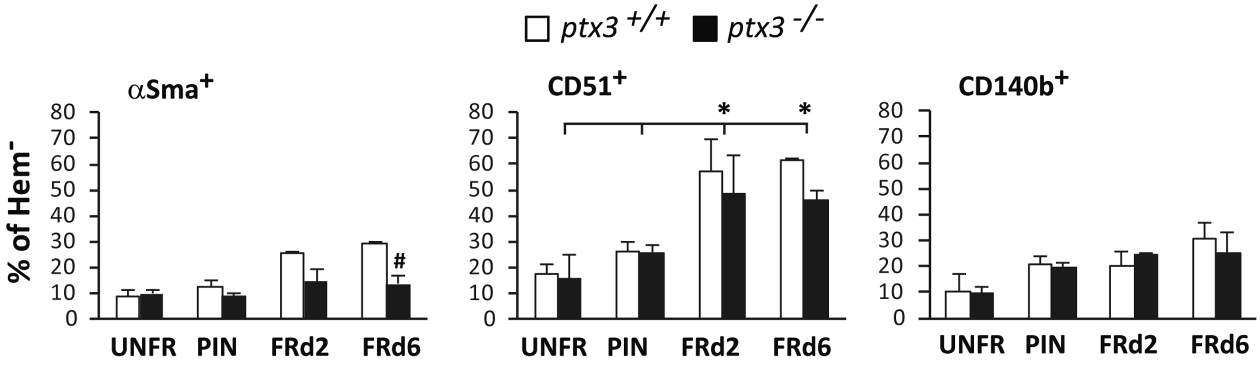

Figure S8. Osteoprogenitor lineage distribution at early post-fracture days in *ptx3*$^{+/+}$and *ptx3*$^{-/-}$ mice on B6 background. A stabilized closed transversal mid-tibial fracture model was applied in female *ptx3*$^{+/+}$and *ptx3*$^{-/-}$ mice (14-16 weeks of age). Callus tissue were harvested from unfractured mice (UNFR), mice with inserted pins (PIN) and mice at 2 (FRd2) and 6 (FRd6) days post-fracture, and analyzed by flow cytometry. Distribution of osteoprogenitor cell subsets (αSma, CD51 or CD140b) within non-hematopoietic/non-endothelial population ($CD45^{-}Ter119^{-}CD31^{-}$) of cells in unfractured tibial periosteal layer (UNFR), periosteal layer of bones with inserted pin (PIN) and fractured areas involving periosteal reaction and callus tissue (FRd2 and FRd6) from *ptx3*$^{+/+}$and *ptx3*$^{-/-}$ mice. Values are presented as mean ± standard deviation (n=6-8 mice per group); statistically significant difference is marked on plots (* $p<0.05$ between time-points within the *ptx3*$^{+/+}$ or *ptx3*$^{-/-}$ groups; # $p<0.05$ between corresponding *ptx3*$^{+/+}$ and *ptx3*$^{-/-}$ groups for the same time-point; ANOVA and Student-Neuman-Keuls post-hoc test).
